# Wavelength-controlled photothermal reduction of carbon dioxide to produce fuels over Nb_2_AlC/TiO_2_ heterostructures in a continuous-flow reactor

**DOI:** 10.1039/d6ra09084b

**Published:** 2026-11-11

**Authors:** Anuradha Sharma, Muhammad Tahir, Naveen Kumar

**Affiliations:** a Chemical and Petroleum Engineering Department, United Arab Emirates University Al Ain 15551 United Arab Emirates muhammad.tahir@uaeu.ac.ae bttahir@yahoo.com; b Department of Chemistry, Maharshi Dayanand University Rohtak 124001 India

## Abstract

A highly conductive Nb_2_AlC MAX phase was coupled with TiO_2_ to construct Nb_2_AlC/TiO_2_ heterostructures and evaluated for CO_2_ photothermal reduction under solar radiations. Incorporation of 10 wt% Nb_2_AlC into TiO_2_ significantly improved the photocatalytic performance with 858.8 and 10.5 µmol g^−1^ h^−1^ of CO and H_2_ respectively with a water–methanol mixture at 150 °C. Using the water–methanol mixture, the CO production rate is 77.7 and 1.7 fold higher than using water and pristine TiO_2_ only. Similarly, the corresponding rates were 3.6 and 1.2 fold higher for H_2_ production. The enhanced photocatalytic activity is attributed to the synergistic interaction between Nb_2_AlC and TiO_2_ which facilitates efficient separation and transfer of charge carriers. The effect of reaction temperature on the distribution of reduction products was investigated in detail. *In situ* DRIFT analysis revealed changes in the interactions of CO_2_ with the catalyst surface at different temperatures and also the intermediate species involved during the photocatalytic reaction. Changes in the CO/H_2_ ratio with increasing temperature indicate that the syngas composition can be effectively regulated by adjusting the reaction temperature over the Nb_2_AlC/TiO_2_ heterostructure. Control experiments under dark conditions at 150 °C produced no detectable products confirming the photo-assisted process rather than thermal conversion. The temperature and irradiation-dependent product distribution demonstrates the potential of Nb_2_AlC/TiO_2_ for controllable CO_2_ conversion under continuous-flow conditions. The material also showed consistent performance throughout five consecutive reuse experiments, with no meaningful decline in product generation. These observations support the use of Nb_2_AlC/TiO_2_ heterostructures for light assisted CO_2_ conversion and for producing syngas with an adjustable CO to H_2_ ratio. These findings highlight the use of a conductive Nb_2_AlC MAX phase as a promising noble-metal-free co-catalyst strategy for thermally assisted CO_2_ conversion.

## Abstract 2

A highly conductive Nb_2_AlC MAX phase was coupled with TiO_2_ to construct Nb_2_AlC/TiO_2_ heterostructures and evaluated for CO_2_ photothermal reduction under solar radiations.

## Graphical abstract

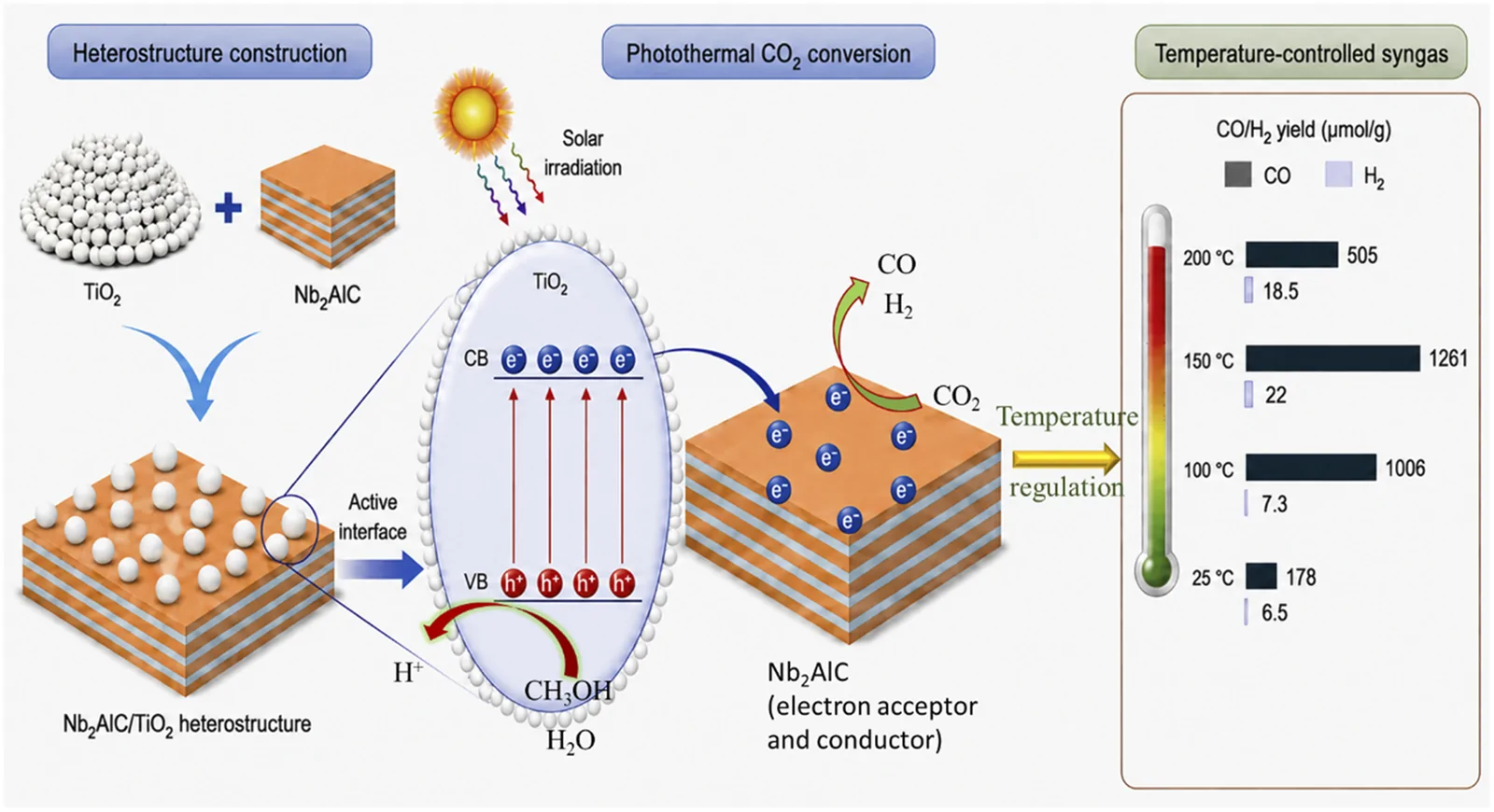

## 1. Introduction

The continuous increase in atmospheric CO_2_ concentration resulting from extensive fossil fuel consumption has become one of the most pressing environmental challenges of the modern era. The accumulation of CO_2_ in the atmosphere contributes significantly to global warming and climate change, motivating the development of sustainable technologies for carbon capture and utilization.^1,2^ The conversion of CO_2_ into value-added fuels and chemicals using renewable energy sources has emerged as an attractive strategy for simultaneously addressing environmental and energy-related concerns.^3^ Among various approaches, solar-driven photocatalytic CO_2_ reduction presents a sustainable carbon-utilization pathway for converting greenhouse gas CO_2_ into value-added fuels and chemical feedstocks.^4^ By coupling solar irradiation with catalytic reactions, CO_2_ can be reduced to carbon monoxide (CO), methane (CH_4_), methanol (CH_3_OH), syngas (CO + H_2_) and other hydrocarbons *via* multi-electron transfer reactions.^5,6^ Photocatalytic CO_2_ reduction has attracted widespread attention as a sustainable route for solar fuel production; still its practical performance remains restricted by two major factors: the inefficient use of available solar spectrum and the inherently slow kinetics of redox reactions taking place on the catalyst surface. As a result, strategies capable of improving both limitations are essential for achieving efficient CO_2_ conversion. Among the available approaches, photothermal catalysis has emerged as an effective alternative because it simultaneously exploits the light and heat components of solar radiation.^7^ Rather than relying solely on photoexcited charge carriers, the additional thermal energy promotes reactant activation, increases rate of interfacial reaction and enhances molecular diffusion. The combined action of thermal energy and photogenerated electrons and holes reduces the energy barrier associated with CO_2_ conversion, leading to improve the overall reaction kinetic.^8^ The cooperative interaction between heating and photochemical excitation not only increases the CO_2_ conversion efficiency but also affects the relative rate of different reaction products. Previous investigations has shown that photothermal CO_2_ hydrogenation can produce CO and multiple hydrocarbons at temperatures considerably below those normally required for conventional thermally driven reactions.^9,10^ Similarly, combining CO_2_ with a sacrificial hydrogen source (such as CH_4_ or H_2_O) under photothermal conditions can also support syngas (CO + H_2_) formation at lower operating temperatures, while reducing the overall energy required for the reaction.^11,12^ Owing to these advantages, photothermal-assisted CO_2_ conversion has become an increasingly important research direction for simultaneously improving catalytic activity and regulating the distribution of resulting reduction products. Continuous-flow photochemistry is emerging in various forms and application areas. Recent studies have demonstrated its potential for rapid visible-light-driven organic reactions,^13^ catalyst recovery and recycling,^14^ and continuous-flow photocatalytic wastewater treatment.^15^ These advances highlight the importance of reactor design and light utilization in developing efficient continuous-flow systems. The application of Nb_2_AlC MAX phase coupled with TiO_2_ for wavelength-controlled photothermal CO_2_ conversion under continuous-flow conditions remains comparatively unexplored. Among the possible products obtained from CO_2_ reduction, syngas is of particular industrial significance because it serves as the primary feedstock for Fischer–Tropsch synthesis and methanol production.^16^ Hydrogen itself is consumed in numerous large-scale chemical and industrial processes.^17^ Nevertheless, obtaining syngas selectively from CO_2_ remains challenging because of the exceptional thermodynamic stability of the CO_2_ molecule and the complicated sequence of coupled electron and proton transfer steps involved in its activation and reduction.^18^ The hydrogen evolution reaction often competes with CO_2_ reduction, making it difficult to control the distribution of CO and H_2_ in the product stream. The development of photocatalytic systems capable of simultaneously enhancing CO_2_ activation and regulating product selectivity (CO *vs.* H_2_, *etc.*) under solar irradiation remains a major research focus. In this context, introducing a photothermal contribution into photocatalytic process provides a promising route for achieving higher conversion rate together with temperature dependent control over syngas composition.^19^ Recent studies have examined a broad range of CO_2_-conversion strategies, including Cu nanoparticles,^20^ molten-salt catalysts and metal–organic/covalent-organic frameworks, highlighting the diversity of material platforms being explored for converting CO_2_ into fuels and value-added chemicals.^21^ Among semiconductor materials investigated for photocatalytic application, TiO_2_ continues to be one of most studied because of its availability, chemical stability, environment compatibility and strong oxidation reduction capability.^22^ Its effectiveness under sunlight is nevertheless limited by its relatively wide band gap of approximately 3.2–3.4 eV. TiO_2_ is activated mainly by the ultraviolet region of the solar spectrum, which represents only a small fraction of the available solar energy. Its performance is further reduced by the rapid recombination of photogenerated electrons and holes before these charge carriers can participate in surface reactions.^23,24^ Together, these limitations significantly reduce its efficiency for CO_2_ photoreduction to different fuels. To overcome these limitations, TiO_2_ is frequently modified by doping, composite or heterostructure formation, and surface sensitization.^23,25^ Such strategies can extend the light absorption into the visible range and provide pathways for efficient charge separation and transfer.^26,27^ Related work on S-scheme/Z-scheme heterojunctions has also investigated interfacial charge separation for solar-driven photoelectrochemical water splitting,^28^ dye degradation,^29^ antibiotic degradation,^30^ herbicides removal,^31^ providing broader context for the role of heterostructure engineering in light-driven catalytic systems in reactions beyond CO_2_ conversion and environmental remediation.

The engineering of reconfigurable neuron-like conductive networks and the understanding of boron interfacial oxidation dynamics are two examples of recent advances in materials science and energy systems.^32–35^ Researchers have increased the strength-ductility synergy of refractory structures by advanced manufacturing, refined thermoelectric generators *via* impinging jet arrays, and developed oxide layer removal processes in high-performance alloys. Customized carbon nitride photocatalysts and innovative hierarchical nanocomposites greatly improve catalytic performance for energy conversion and environmental remediation.^36–39^ Other than these systems, advanced lattice density functional theory models clarify gas adsorption behaviors in complex nanoporous media, sustainable carbonation processes valorize industrial byproducts like red mud, and heteroatom doping strategies drive effective bifunctional oxygen electrocatalysis.^40,41^

Three-dimensional transition metal MAX and 2D MXene structures have emerged as highly promising cocatalysts for TiO_2_. These materials possess metallic conductivity, layered structure, and abundant surface functional groups, making them excellent electron sink that promote charge separation in TiO_2_ composites. Chen *et al.*^42^ reported TiO_2_ nanoparticles grown *in situ* on Ti_3_C_2_ MXene form an intimate interface with a Schottky junction which delivered CO and CH_4_ production rates 2.8 and 4.0 times higher than that of bare TiO_2_, respectively. V_2_AlC incorporated into porous g-C_3_N_4_ produced CO/H_2_ syngas at rates 2.2 and 1.99 times higher than g-C_3_N_4_ alone, owing to the role of MAX phase in promoting photocarriers separation.^43^ Mechanistically, these improvements arise because the strong electronic coupling at the TiO_2_ metal carbide interface that drives photogenerated electrons into the conductive MAX/MXene layer, resulting in suppressing electron–hole recombination. In particular, niobium-based carbides are notable for their excellent electron trapping ability. Barsoum *et al.*^44^ report that Nb_2_AlC is a metallic conductor with room-temperature conductivity of the order of 3.5 × 10^−4^ S cm^−1^) with low thermal expansion, stabile up to 1000 °C, and exhibit localized surface plasmon resonances (LSPR) effects that generate energetic charge carriers and suppressing recombination. Nb_2_AlC derived Nb_2_C MXene has been used for CO_2_ conversion *via* photothermal catalysis.^45^ Due to its high work function it can accumulate electrons more effectively than many other cocatalysts, yielding superior photocatalytic activity. These findings align with the intrinsic properties of the Nb_2_AlC MAX phase. Carbide-based materials and MXenes can enhance light-assisted CO_2_ conversion through complementary effects like CO_2_ adsorption, interfacial charge transfer, and photothermal activation. Ti_3_C_2_T_*x*_-based TiO_2_ heterostructures have demonstrated enhanced selective CO_2_-to-CO conversion demonstrating the synergistic contribution of photothermal heating and interfacial charge migration.^46,47^ Incorporating Ti_3_C_2_ MXene with nitrogen-doped TiO_2_ enhanced photocatalytic CO_2_ reduction through improved CO_2_ adsorption and charge separation at the TiO_2_/MXene interface.^48^ Another study demonstrated CsPbBr_3_/Ti_2_N an MXene-supported semiconductor system where partial oxidation produces TiO_2_ moieties on the Ti_2_N surface could efficiently utilize photothermal effects for CO_2_ conversion, with reaction temperature influencing the distribution of carbon-containing products.^49^ Nb_2_C-supported Cu and Mo_2_C/TiO_2_ systems have also demonstrated the potential of transition-metal carbides for interfacial electronic interactions and CO_2_ conversion, including continuous-flow operation.^50,51^ These studies collectively highlight the multifunctional role of conductive carbide-based materials in light-assisted CO_2_ conversion including charge transport, photothermal energy utilization, and reactant activation. Despite these advances for CO_2_ conversion, the direct integration of intact Nb_2_AlC MAX phase with TiO_2_ for photothermal CO_2_ conversion remains comparatively unexplored. Nb_2_AlC can be further explored in photocatalytic applications. Nb_2_AlC/TiO_2_ composite can accelerate charge transport by leveraging exceptional electron conductivity and thermal robustness of Nb_2_AlC and making it a promising component for TiO_2_-based photothermal CO_2_ conversion.

In this work, we hypothesized that combining Nb_2_AlC with TiO_2_ could improve the performance of TiO_2_ during photothermal CO_2_ conversion. The conductive nature of Nb_2_AlC may assist in the transport of photogenerated electrons, while its thermal response could contribute to the activation of surface reactions. As a result, the Nb_2_AlC/TiO_2_ composite was expected to promote CO_2_ reduction and influence the relative formation of CO and H_2_. To examine this hypothesis, composites containing different amounts of Nb_2_AlC were prepared and tested in a continuous-flow reactor. The simple ultrasonication-assisted physical-mixing method used here avoids chemical etching and complicated deposition steps, which may facilitate scale-up. As a noble-metal-free conductive component, Nb_2_AlC may reduce dependence on expensive Pt, Au, or Pd-based cocatalysts. The photocatalytic reactions were conducted from 25 °C to 200 °C under solar-irradiations in the presence of methanol as a sacrificial agent where CO and H_2_ were measured as the gaseous products. This approach allows the influence of photothermal activation on CO formation and competitive H_2_ evolution to be examined, providing insight into the temperature dependent regulation of the CO/H_2_ ratio. The effects of Nb_2_AlC loading, reaction temperature, sacrificial-agent, and irradiation wavelength on CO and H_2_ formation were systematically investigated. *In situ* DRIFT spectroscopy was employed to examine the interaction of CO_2_ with the catalyst surface and the evolution of surface intermediates at different temperatures. Cyclic stability was examined for consecutive five cycles where photocatalyst exhibited excellent stability. A reaction mechanism was proposed to clearly understand the reaction process.

## 2. Experimental

This section describes the synthesis of Nb_2_AlC/TiO_2_ composites, various characterization techniques to investigate the structural, morphological, optical, and electrochemical properties of the materials, and photoactivity procedure evaluated in a continuous-flow reactor under solar irradiation to examine the effects of Nb_2_AlC loading, reaction temperature, feed composition, and irradiation wavelength.

### 2.1 Chemicals

Chemicals used for the synthesis of photocatalytic materials were TiO_2_ (titanium(iv) oxide, anatase, 99%, Aldrich), Nb_2_AlC MAX (AR grade), methanol (99.9%, Merck).

### 2.2 Synthesis of Nb_2_AlC/TiO_2_ composite

Nb_2_AlC/TiO_2_ composites were prepared by simple ultrasonication and physical mixing. TiO_2_ (titanium(iv) oxide, anatase, 99%, Aldrich), and Nb_2_AlC MAX were separately dispersed in methanol (99.9%, Merck) under ultrasonication. In particular procedure for 10% Nb_2_AlC/TiO_2_ composite, 0.5 g TiO_2_ taken in 30 ml methanol and 0.05 g Nb_2_AlC in 10 ml methanol were ultrasonicated for 60 min in separate beakers. Then, the MAX solution was added slowly into TiO_2_ solution and kept under constant magnetic stirring for three hours. Thereafter, the solution was dried overnight in an oven at 100 °C. Again, the product was crushed and obtained the final material labelled as 10-Nb_2_AlC/TiO_2_. Similar procedure was followed to prepare different compositions (5%, 10%, and 15%) by adding the calculated amounts of MAX phase into TiO_2_ and materials were labelled as 5- Nb_2_AlC/TiO_2_, 10-Nb_2_AlC/TiO_2_, and 15-Nb_2_AlC/TiO_2_. This preparation method was selected to establish close physical contact between Nb_2_AlC and TiO_2_ while preserving the structure of the Nb_2_AlC MAX phase (Fig. 1).

**Fig. 1:**
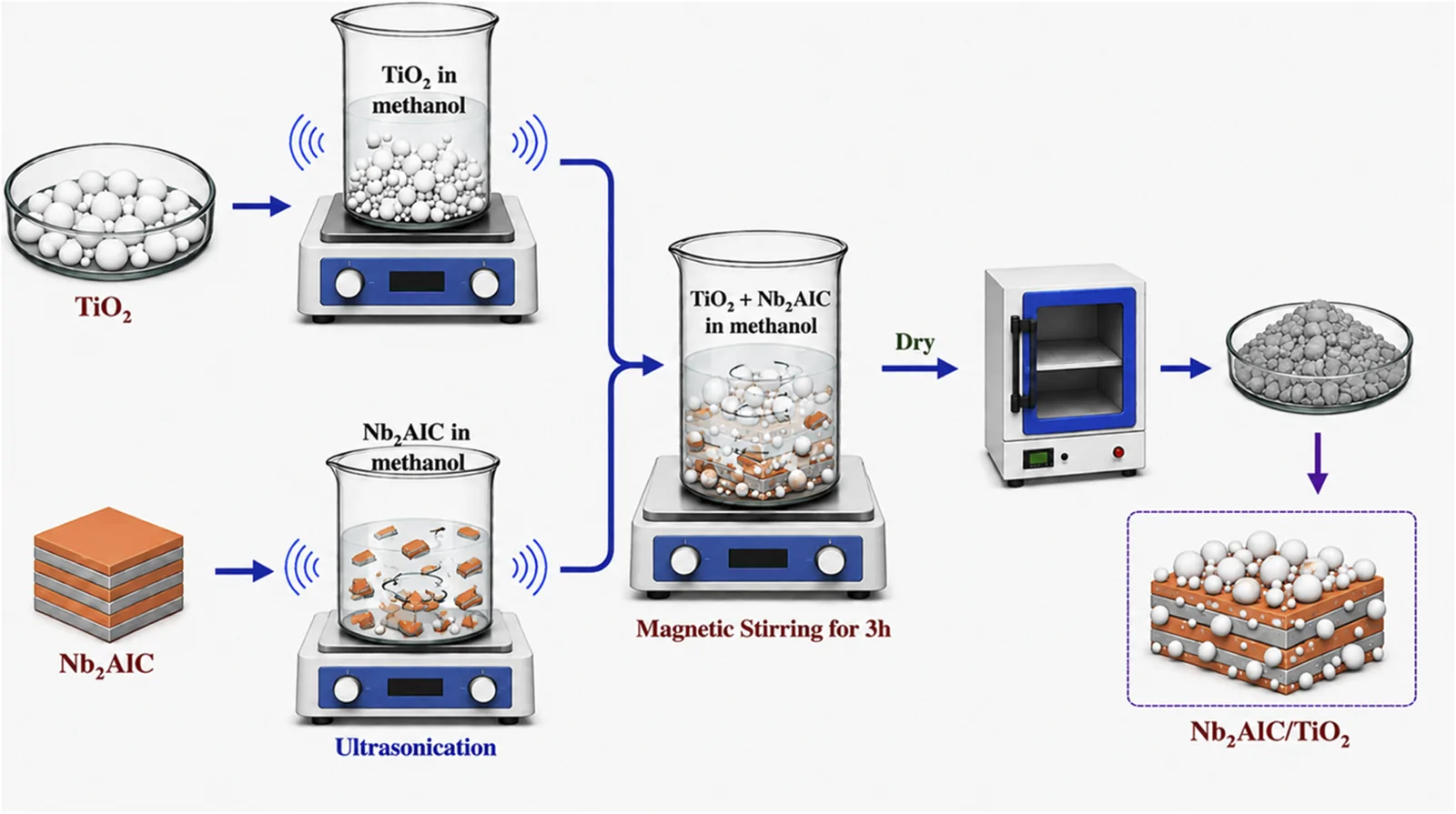
A schematic overview of the synthesis process for Nb_2_AlC/TiO_2_ composites.

### 2.3 Characterization

The synthesized materials were characterized using various analytical techniques to evaluate their structural, morphological, optical, and electrochemical properties. The crystalline phase and structural features were investigated through X-ray diffractometer (Malvern Panalytical X'Pert diffractometer, Netherlands). FTIR (JASCO FTIR 4700, United Kingdom) was used to investigate surface functional groups of the catalyst. Surface morphology was examined by Scanning electron microscopy (JSM-IT210 and DII-29030SCTR smart Coater). High-resolution transmission electron microscopy (HRTEM) was employed to investigate the microstructures and examine the interfacial contact (Thermofisher Talos F200i).

UV-visible diffuse reflectance spectra were obtained using JASCO (V-670) instrument to examine the band gap positions and optical absorption behavior of the synthesized materials. Photoluminescence (PL) measurements were performed by using a (NOST FEX-u, South Korea) spectrophotometer operated at the excitation wavelength of 345 nm. XPS analysis was done on (Thermo Nexsa G2 X-ray Photoelectron Spectrometer) to examine the surface chemical states of the elements in the materials. Electrochemical measurements including Electrochemical Impedance Spectroscopy (EIS) and Cyclic Voltammetry (CV) were done in a three electrode arrangement and Wuhan Corrtest Instruments Corp. Ltd CS350 workstation. Ag/AgCl served as reference electrode, Pt mesh was the counter electrode, and the catalyst-coated electrode as the working electrode. Aqueous 3 M KOH solution was used as the electrolyte. For the preparation of working electrode, photocatalyst was taken as active material, activated carbon, and polyvinylidene fluoride (PVDF) binder in a ratio of (85 : 10 : 5) and mixed in *N*-methyl-2-pyrrolidone (NMP). The resulting homogeneous slurry was coated uniformly (1 × 1 cm^2^) on carbon cloth piece and dried at 80 °C prior to electrochemical measurements.

### 2.4 Photoactivity test

The photocatalytic CO_2_ reduction performance of the prepared materials was examined in a continuous fixed bed photoreactor system. The continuous-flow configuration was selected to continuously remove gaseous products from the reactor and to evaluate the catalyst under conditions relevant to practical CO_2_ conversion. In the procedure, 0.1 g of the catalyst was uniformly distributed over a fixed bed prepared from quartz wool and placed at the bottom of the reaction chamber. It consisted of a quartz cylindrical vessel and fitted with light source (Xenon lamp, intensity = 100 mW cm^−2^) located over the quartz glass window which allows light illumination over the fixed bed of the photocatalytic material. Before starting the experiments, the gaseous feed of CO_2_ was passed through a water saturator to either carry water or water–methanol with CO_2_ before entering into the reaction chamber. Mass flow controller was fitted before the water saturator to control the gaseous flow rate. Now, before light illumination, the reaction bed was saturated for 30 min with the continuous flow of the 5 ml min^−1^ of feed gas mixture. After saturation of the material, light source was positioned over so as to cover the reactor vessel completely. For photothermal experiments, the desired reaction temperature (25 °C to 200 °C) was achieved using a bottom-mounted heater that directly measures the temperature over the catalyst surface with the thermocouple inserted inside the reaction chamber. Now the products of the reaction were monitored with the help of an online gas chromatograph (Inficon Micro GC Fusion) which was directly coupled with the reactor outlet. The products from the photocatalytic reaction were quantified at every 20 min interval for 2 h.

Apparent quantum efficiency (AQE) was further calculated to evaluate the light-utilization efficiency of the catalysts. The AQE was estimated based on an incident irradiance of 1 sun (100 mW cm^−2^), an illuminated area of 12.56 cm^2^, and an effective irradiation wavelength of 420 nm. The incident optical power was calculated from *P* × *S*, where *P* represents the incident irradiance (100 mW cm^−2^) and *S* is the illuminated area (12.56 cm^2^), giving a total incident optical power of 1.25 W. The corresponding photon flux was then estimated from the photon energy using eqn (1) and considering two electrons for the formation of each CO and H_2_ molecule, the AQE was calculated according to eqn (2).1
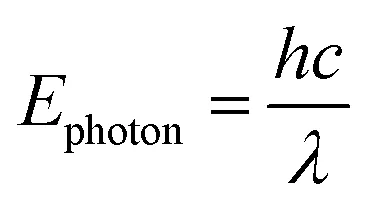
2
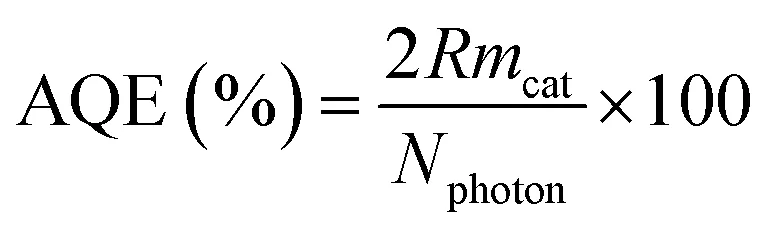
*R* is the product evolution rate (µmol g^−1^ h^−1^), *m*_cat_ is the catalyst mass (0.1 g), and *N*_photon_ is the incident photon flux (µmol photons h^−1^).

## 3. Results and discussion

This section presents a comprehensive investigation of the structural, morphological, optical, electrical, and photocatalytic properties of the Nb_2_AlC/TiO_2_ composites for photothermal CO_2_ conversion under continuous-flow conditions. The influence of Nb_2_AlC incorporation on the physicochemical properties and catalytic performance of TiO_2_ is systematically evaluated, with particular emphasis on CO and H_2_ production, reaction temperature, light irradiation, and the role of the sacrificial agent. Furthermore, mechanistic insights into the CO_2_ conversion pathway are explored through diffuse reflectance infrared Fourier transform (DRIFT) spectroscopy, while the stability of the optimized composite and the tunability of product selectivity are examined to assess its potential for efficient and sustainable CO_2_ reduction.

### 3.1 Material's characterization

The crystalline phase composition and structural characteristics of the synthesized Nb_2_AlC/TiO_2_ composites were investigated using X-ray diffraction (XRD) as shown in Fig. 2a. The diffraction patterns of pristine TiO_2_ exhibited well defined reflections located at 2*θ* values of 25.27°, 37.85°, 48.01°, 53.89°, 55.01°, and 62.62°, which can be indexed to the (101), (004), (200), (105), (211), and (204) crystallographic planes of anatase TiO_2_, respectively [JCPDS 21-1272]. The XRD pattern of Nb_2_AlC displays two main characteristic reflections at 12.95° and 38.98° corresponding to the (002) and (102) planes respectively. The other peaks at 26.09°, 52.29°, 70.62°, and 73.59° correspond to the (004), (105), (109), and (116) planes of the layered hexagonal MAX phase structure (space group *P*6/*mmc*) [JCPDF #00-030-0033].^52,53^ In the composite 10-Nb_2_AlC/TiO_2_, the diffraction peaks of both the components were observed indicating successful integration of Nb_2_AlC into TiO_2_. No extra peaks other than these two compounds are observed in composite confirming the preserved crystal structure of both the components and no structural transformation of either phase was observed.

FTIR spectra of all samples were employed to elucidate the surface functional groups and chemical bonding interactions in the materials. The spectra were recorded within the spectral range of 400–4000 cm^−1^ as shown in the Fig. 2b. Pure TiO_2_ exhibited a broad and dominant absorption band centered at 616 cm^−1^ and extended from 450–700 cm^−1^ is attributed to the stretching vibrational modes of Ti–O–Ti and O–Ti–O bonds which are typical characteristic of the anatase TiO_2_ lattice.^54^ The absorption band observed around 3446 cm^−1^ correspond to the O–H stretching vibrations from surface hydroxyl groups and adsorbed water molecules.^55^ Nb_2_AlC MAX phase gives a much weaker FTIR response, as expected for its mixed metallic ceramic nature. Nb_2_AlC/TiO_2_ composite spectrum essentially superimposes TiO_2_ and Nb_2_AlC vibrations, but slight shifts in peak positions and changes in band intensities compared to the individual components suggest interfacial interactions between TiO_2_ and Nb_2_AlC. The Ti–O–Ti vibrational band (450–700 cm^−1^) remains present at the similar position. A bending mode of adsorbed H_2_O or Ti–OH bond appears near 1636 cm^−1^ in the composite material. The broad O–H stretching region around 3446 cm^−1^ becomes more pronounced indicating a substantially higher density of surface hydroxyl groups and adsorbed moisture.

**Fig. 2:**
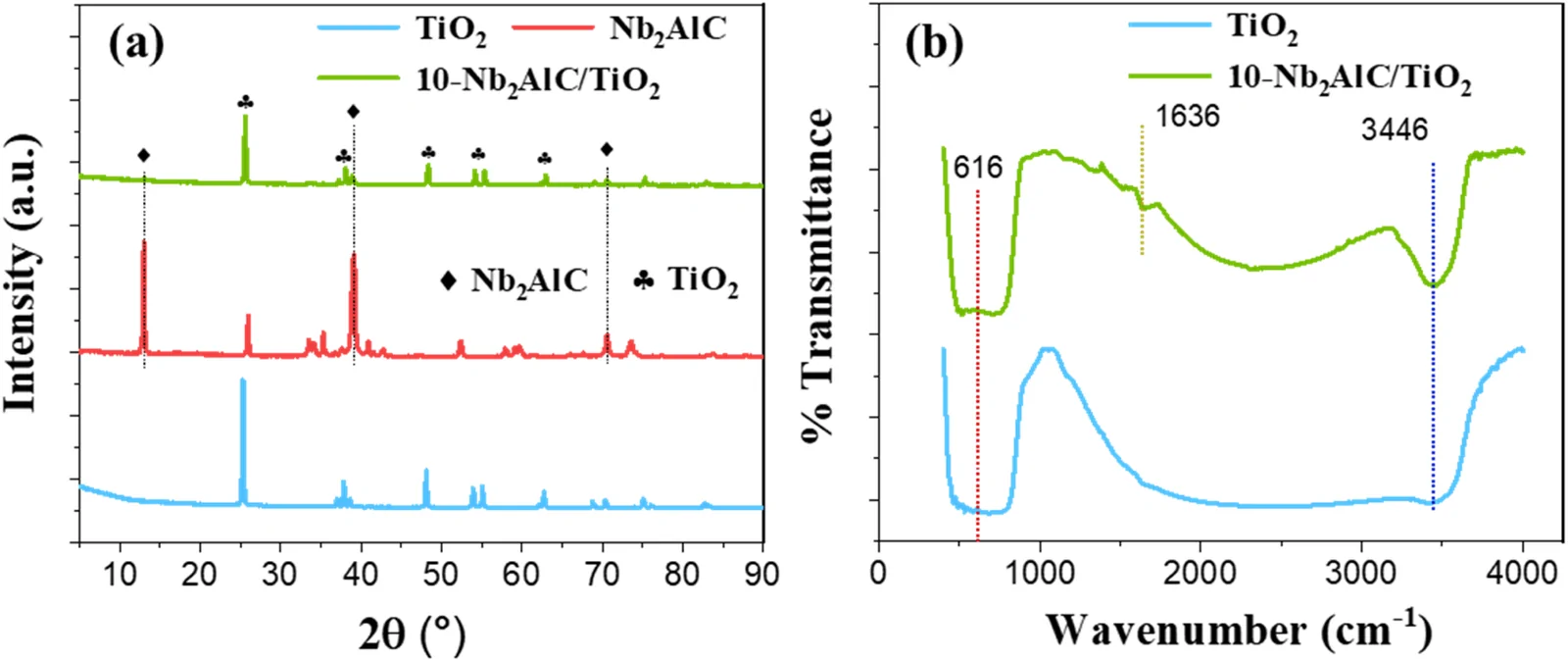
(a) XRD patterns and (b) FTIR spectra of pristine TiO_2_, Nb_2_AlC MAX phase and the 10-Nb_2_AlC/TiO_2_ heterostructure.

The morphology of the pristine materials and the synthesized Nb_2_AlC/TiO_2_ composite was examined using SEM and HRTEM, while elemental mapping and EDS were employed to verify the elemental distribution and composition. As shown in Fig. 3a, pristine TiO_2_ consists of nearly spherical nanoparticles that are densely packed, forming agglomerated clusters because of their high surface energy. In contrast, the SEM image of pristine Nb_2_AlC (Fig. 3b) exhibits a typical layered morphology composed of stacked plate-like sheets with smooth surfaces, which is characteristic of MAX phase materials. After incorporating Nb_2_AlC into TiO_2_ through the ultrasonication-assisted physical mixing process, the composite (Fig. 3c) retains the layered framework of Nb_2_AlC while TiO_2_ nanoparticles are uniformly anchored onto its surface. The intimate contact between the two constituents suggests the successful formation of a heterostructure.

**Fig. 3:**
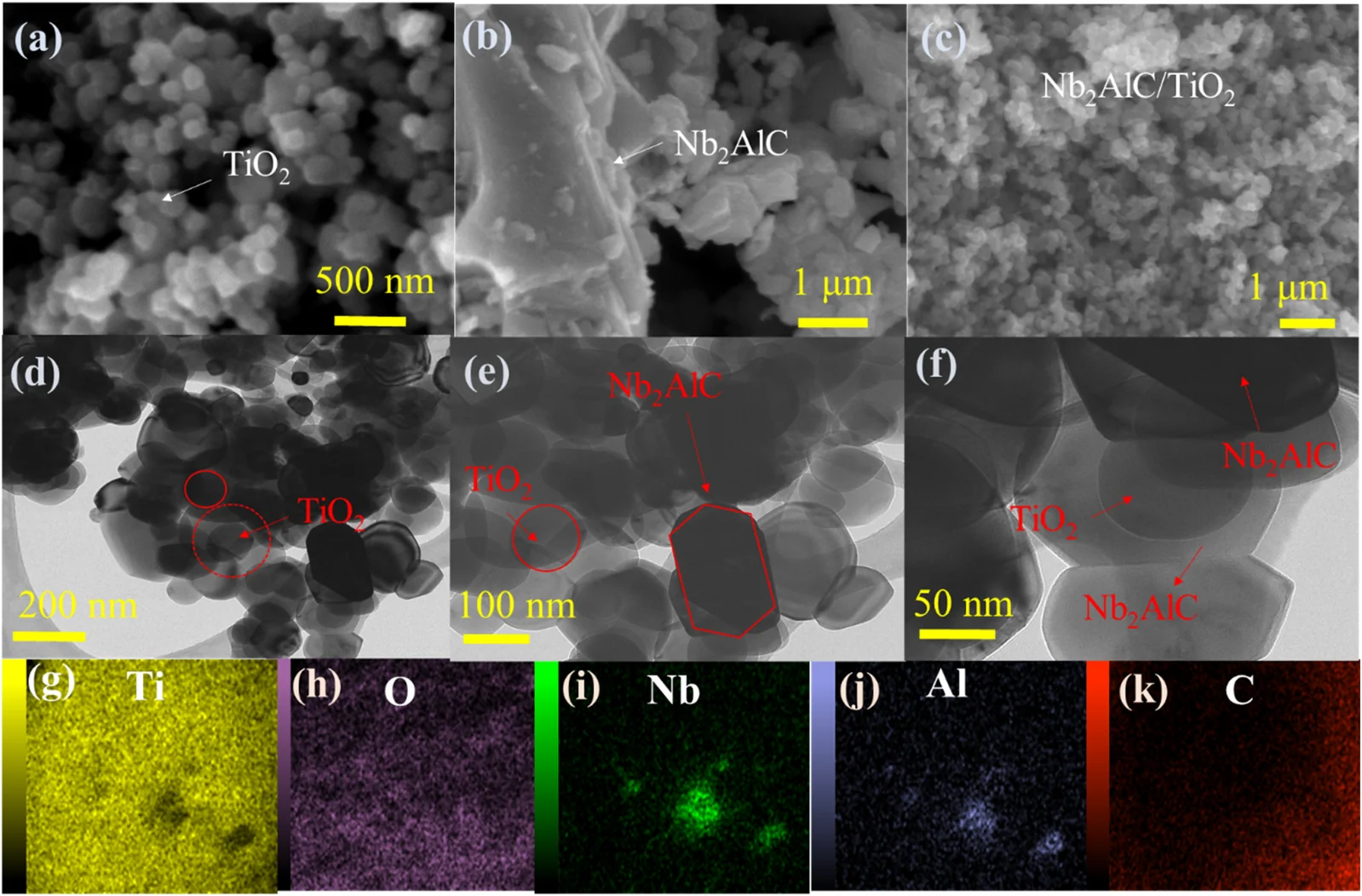
SEM images of (a) pristine TiO_2_, (b) pristine Nb_2_AlC, (c) 10-Nb_2_AlC/TiO_2_ composite, (d–f) TEM images, (g–k) elemental mapping of Ti, O, Nb, Al, and C of the 10-Nb_2_AlC/TiO_2_ composite.

The TEM images provide further insight into the microstructure of the composite. The images were analyzed to identify the contact region between the two components. The observed contact between Nb_2_AlC and TiO_2_ provides additional structural evidence of interfacial contact within the composite. As illustrated in Fig. 3d–f, the thin Nb_2_AlC sheets serve as a supporting matrix for the TiO_2_ nanoparticles, which are well dispersed over the surface rather than forming large isolated agglomerates. Such homogeneous decoration increases the interfacial contact between TiO_2_ and Nb_2_AlC, creating abundant heterointerface that are expected to facilitate charge transport across the composite. The close interaction between the semiconductor nanoparticles and the conductive MAX phase is advantageous for suppressing charge recombination during photocatalytic reactions. The elemental distribution within the composite was investigated by the EDS elemental mapping shown in the Fig. 3g–k. The mapping images clearly reveal the presence of Ti, O, Nb, Al, and C throughout the selected region. Ti and O are distributed uniformly over the entire area, while Nb and Al are localized within the layered domains of Nb_2_AlC. Carbon is also detected across the composite, consistent with the carbon layers of the MAX phase. The absence of obvious elemental segregation indicates that the ultrasonication-assisted mixing process promotes a homogeneous dispersion of Nb_2_AlC within the TiO_2_ matrix.

The corresponding EDS (Fig. S1) spectrum further confirms the coexistence of Ti, O, Nb, Al, and C in the synthesized composite. The measured elemental composition agrees well with the expected constituents of the Nb_2_AlC/TiO_2_ heterostructure, providing additional evidence for the successful integration of the two materials. The combined SEM, TEM, and EDS analyses demonstrate that TiO_2_ nanoparticles are effectively immobilized on the layered Nb_2_AlC support, resulting in a well-integrated composite architecture that is favorable for efficient interfacial charge transfer and improved photocatalytic performance.

The optical absorption characteristics of all the materials were examined by UV-visible diffuse reflectance spectroscopy and observed spectra are shown in the Fig. 4a. Pure TiO_2_ exhibited strong absorption in the ultraviolet region and a sharp absorption edge near 380 nm which is characteristic for anatase TiO_2_. The Nb_2_AlC displayed a continuous, broad and high intensity absorption throughout the complete wavelength range. This is due to its metallic nature and unique layered electronic structure which possess partially filled d-bands and free charge carriers.^56^ Such broadband absorption suggests that Nb_2_AlC can act as an effective light absorber and electron transport medium when coupled with semiconductor photocatalysts. After incorporation of Nb_2_AlC into TiO_2_, the composite retained the characteristic absorption behavior of TiO_2_, and also increase in absorption intensity relative to TiO_2_ was observed. Although the absorption intensity of the composite remained lower than that of pure Nb_2_AlC, its stronger absorbance compared with TiO_2_ including in the ultraviolet region, indicates that the Nb_2_AlC phase contributes to improved light harvesting within the heterostructure. The intimate contact between the two phases is expected to improve the utilization of incident photons.^57^ In this heterostructure, Nb_2_AlC serves as a highly conductive route through which photogenerated electrons can migrate, thereby limiting their recombination with holes. As a result, more charge carriers remain available to participate in the CO_2_ reduction reaction, leading to enhanced photocatalytic performance owing to increased photon absorption in the composite heterostructure than pristine TiO_2_. To quantitatively evaluate the optical band gap energy using the Tauc plot, the Kubelka–Munk function was employed.^58,59^ The calculated values of band gap energies for TiO_2_ and 10-Nb_2_AlC/TiO_2_ were 3.38 and 3.34 eV, respectively as shown in the Fig. 4b.

**Fig. 4:**
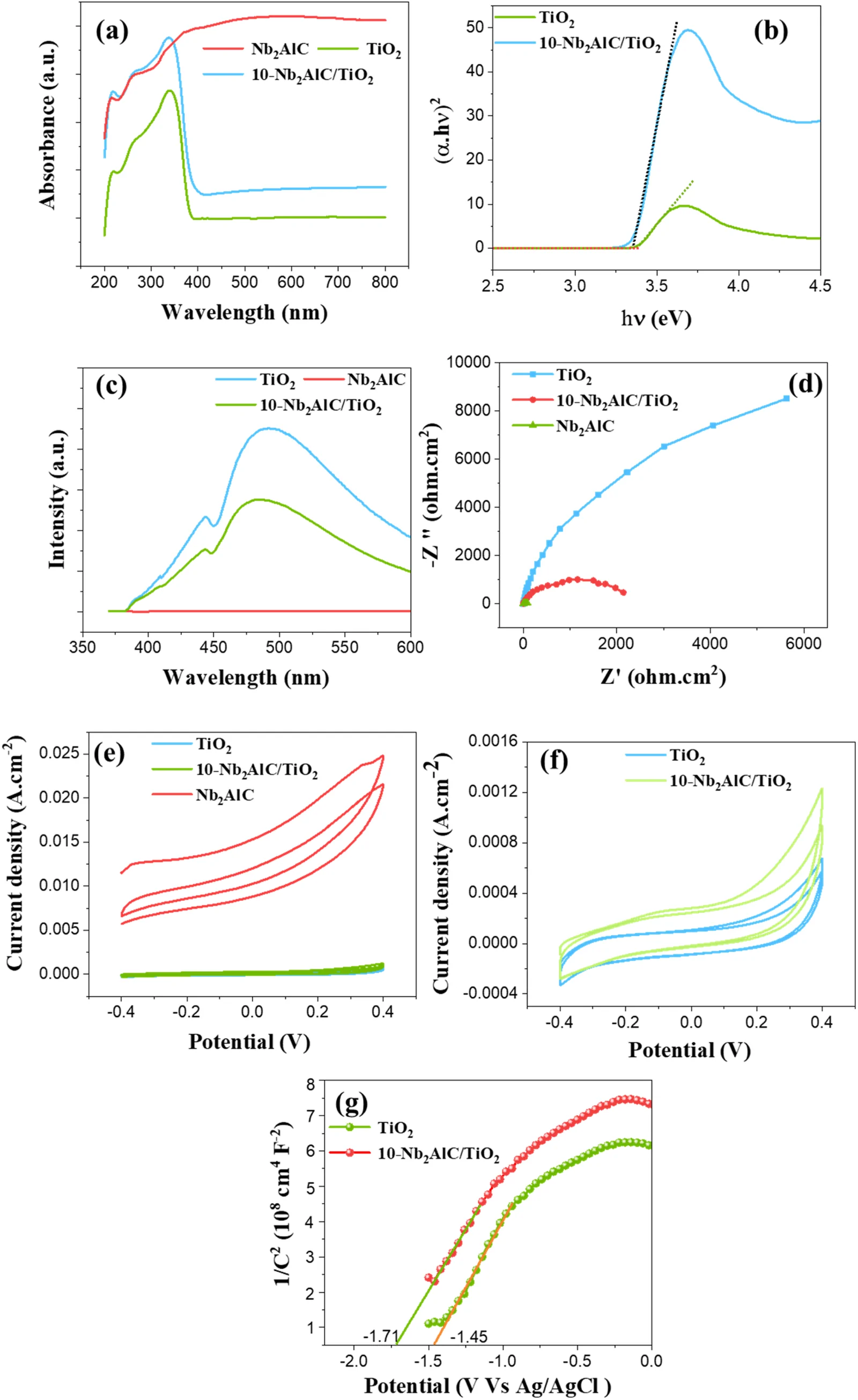
(a) UV-Vis DRS, (b) optical band gap analysis by Tauc plot, (c) PL analysis, (d) EIS, (e and f) CV, (g) Mott–Schottky plot of TiO_2_, Nb_2_AlC, and 10-Nb_2_AlC/TiO_2_ samples.

PL spectroscopy was employed to investigate the migration, transfer, and recombination dynamics of photogenerated electron–hole pairs. Fig. 4c compares the PL spectra of pristine TiO_2_, Nb_2_AlC, and the 10-Nb_2_AlC/TiO_2_ composite taken at excitation wavelength 345 nm. The PL spectrum of pure TiO_2_ exhibits a strong and broad emission band centered around 500 nm which is attributed to radiative recombination of photoinduced electrons and holes through intrinsic defect states, such as oxygen vacancies and surface traps within band structure of TiO_2_.^60,61^ This intense emission signifies a high rate of charge carrier recombination, which inherently limits photocatalytic efficiency. The Nb_2_AlC displays a virtually flat and negligible PL response. This absence of luminescence is attributed to its metallic MAX phase and its conductive nature. The conductive layered structure of Nb_2_AlC facilitates rapid electron migration and acts as an electron-accepting medium, suppressing the direct recombination of photogenerated electron–hole pairs in TiO_2_. 10-Nb_2_AlC/TiO_2_ composite shows a substantially lower emission intensity relative to pure TiO_2_ throughout the entire spectral region. This decline in PL emission in the composite may indicate reduced radiative recombination of photogenerated charge carriers, but PL quenching can also arise from photon absorption by the dark Nb_2_AlC powder. The PL result alone does not establish the direction of interfacial electron transfer. To further examine the charge-transfer behavior, electrochemical impedance spectroscopy (EIS) was conducted. The Nyquist plots shown in Fig. 4d reveal a significant difference in impedance behavior among TiO_2_, Nb_2_AlC, and the 10-Nb_2_AlC/TiO_2_ composite. Generally, the diameter of the semicircle in the high-frequency region is associated with the charge-transfer resistance at the electrode/electrolyte interface. Pure TiO_2_ exhibits the largest semicircular arc, indicating a relatively high charge transfer resistance and less electron transport. Such behavior is commonly observed in semiconductor photocatalysts because of their limited electrical conductivity and rapid charge recombination tendency.^62^ In contrast, Nb_2_AlC displays a substantially lower and negligible impedance response, reflecting its highly conductive MAX phase structure and efficient electron transport capability. Notably, the 10-Nb_2_AlC/TiO_2_ composite exhibits a much smaller semicircle diameter than pristine TiO_2_, demonstrating a pronounced reduction in charge-transfer resistance.

The electrochemical charge storage behavior and charge transfer characteristics of TiO_2_, Nb_2_AlC, and 10-Nb_2_AlC/TiO_2_ composite were investigated by cyclic voltammetry in 3 M KOH electrolyte using a three electrode configuration with scan rate 50 mV s^−1^. As shown in the Fig. 4e, Nb_2_AlC displays the highest current density and the largest enclosed CV area within the investigated potential range indicating its superior metallic conductivity and its ability to support rapid electron transport. This behavior originates from the metallic nature of the MAX phase which provides the pathway for rapid electron transport during the electrochemical process. A noticeable improvement is also observed after incorporating Nb_2_AlC into TiO_2_. As presented in Fig. 4f, the 10-Nb_2_AlC/TiO_2_ composite deliver a high current response and a larger CV area than pristine TiO_2_, suggesting that the conductive Nb_2_AlC phase facilitates electron movement throughout the composite matrix.^63^ The obtained CV results are consistent with the EIS analysis, suggesting that the incorporation of Nb_2_AlC promotes charge transport and suppresses electron–hole recombination, thereby improving the utilization of photogenerated charge carriers during photocatalysis.

The Mott–Schottky analysis was done to evaluate flat band potentials (*E*_FB_) of TiO_2_ and 10-Nb_2_AlC/TiO_2_ at 1000 Hz provided in Fig. 4g. 0.1 M KCl solution was used as the electrolyte during analysis. Both the materials exhibited positive slopes validating n-type semiconductor nature of the materials. The *E*_FB_ values for TiO_2_ and 10-Nb_2_AlC/TiO_2_ were found −1.45 and −1.71 eV (*vs.* Ag/AgCl), respectively. Flat band potential is considered typically 0.2 V positive relative to the conduction band position (*E*_CB_) in n-type semiconductors,^64^ accordingly the calculated values of *E*_CB_ are −1.65 and −1.91 eV for TiO_2_ and 10-Nb_2_AlC/TiO_2_, respectively. As we already calculated values of band gap energies using Kubelka–Munk function, the calculated values for *E*_VB_ are +1.73 and +1.43 eV for TiO_2_ and 10-Nb_2_AlC/TiO_2_, respectively. The calculated band positions shown more negative conduction band potential in the composite than pure TiO_2_ suggesting enhanced reducing power of the photogenerated electrons and a potentially greater thermodynamic driving force for CO_2_ reduction, which is consistent with the enhanced CO formation observed for the composite.

XPS was employed to examine the elemental composition of the catalyst surface together with the corresponding chemical states of the constituent elements. As illustrated in Fig. 5a, the survey spectrum confirms the existence of Ti, O, Nb, Al, and C, demonstrating the successful formation of the Nb_2_AlC/TiO_2_ composite. The high-resolution Ti 2p spectrum (Fig. 5b) consists of well-defined photoelectron peaks located at 458.5 eV and 464.2 eV, which are attributed to the Ti 2p_3/2_ and Ti 2p_1/2_ spin orbit components respectively. The measured energy difference of approximately 5.7 eV agrees well with the reported value for Ti^+4^ species and confirms that titanium is predominantly present in the tetragonal TiO_2_ lattice, consistent with previous reports.^65^ The deconvoluted O 1s spectrum (Fig. 5c) reveals two chemically distinct oxygen species. The intense sharp peak positioned at 529.8 eV originates from lattice oxygen associated with Ti–O bonds, indicate oxide framework of TiO_2_. In contrast, higher binding energy feature observed at 531.5 eV is assigned to surface oxygen species, including hydroxyl groups and defect-related oxygen adsorbed on the catalyst surface, in agreement with earlier studies.^22^ The high resolution Nb 3d spectrum (Fig. 5d) displays a characteristic spin–orbit doublet with peaks located at 206.8 eV and 209.5 eV corresponding to 3d_5/2_ and 3d_3/2_ states, respectively.^66^ The Al 2p region reveals a broad and singular peak centered at 75.5 eV (Fig. 5e), which confirm the presence of Al^+3^ associated with the Nb_2_AlC phase. The deconvoluted C 1s spectrum (Fig. 5f) displays three distinct carbon environments. The primary peak at 284.5 eV is associated with C–C bonds and subordinate peaks emerging at 285.6 eV and 288.5 eV correlate to oxygen functionalized carbon species, specifically C–O and C=O bonds, respectively. These functionalities are typical for surface adsorbed organic contaminants and do not disrupt the bulk phase purity of the composite.^67^ A separate Nb–C component in the expected 281–282 eV region was not clearly resolved from the baseline noise. This may be due to the relatively low Nb_2_AlC loading, the surface-sensitive sampling depth of XPS, and the stronger contribution of surface carbon species in the composite.

**Fig. 5:**
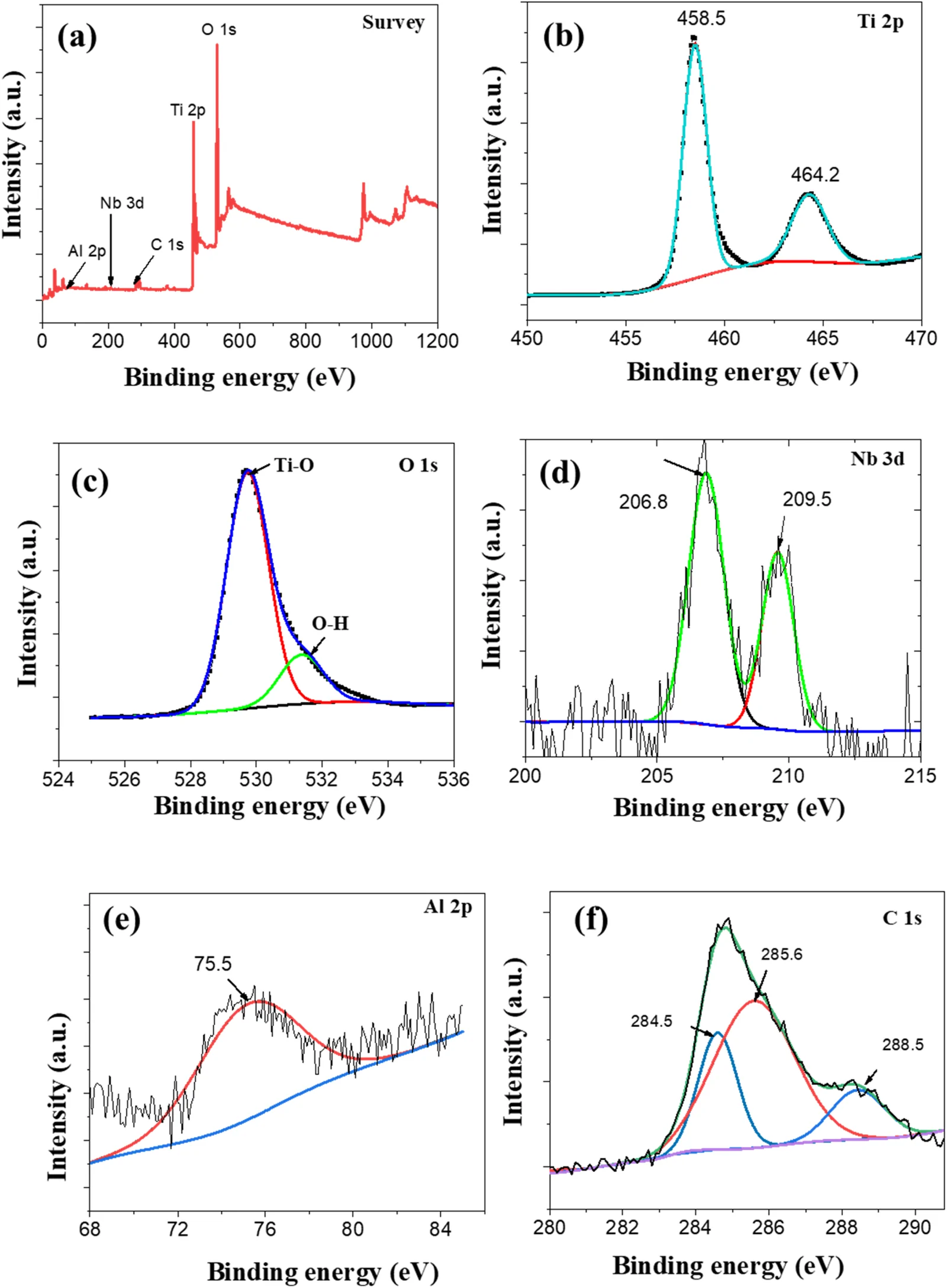
XPS analysis of the 10-Nb_2_AlC/TiO_2_ composite: (a) survey spectrum, (b) Ti 2p spectrum, (c) O 1s spectrum, (d) Nb 3d spectrum, (e) Al 2p spectrum, and (f) C 1s spectrum.

### 3.2 Photocatalytic CO_2_ reduction

The photocatalytic performance of TiO_2_ and Nb_2_AlC/TiO_2_ composites was evaluated using a CO_2_–H_2_O–CH_3_OH feed mixture under solar irradiation, with CO and H_2_ monitored as the gaseous products. To assess the effects of the reaction atmosphere and catalyst, control experiments were conducted at 150 °C by replacing CO_2_ with N_2_ and by omitting the catalyst while retaining the water–methanol mixture. A separate experiment without illumination was also conducted to examine product formation under dark conditions at 150 °C. These control results are discussed below alongside the temperature-dependent activity in Section 3.2.3.

#### 3.2.1 Effect of Nb_2_AlC loading

Different amounts of Nb_2_AlC (5, 10, and 15%) were loaded on TiO_2_ and evaluated for the photocatalytic CO_2_ reduction with (CO_2_ + H_2_O + CH_3_OH) as feed mixture at 100 °C under solar radiations. Fig. 6a presents the influence of Nb_2_AlC loadings monitored for the formation of CO and H_2_ during CO_2_ photoreduction. Pristine TiO_2_ produced 333 and 3.6 µmol g^−1^ h^−1^ of CO and H_2_ respectively under studied reaction conditions. Upon incorporation of Nb_2_AlC into TiO_2_ a gradual enhancement in photocatalytic activity was observed for both the gaseous products up to 10% loading amount of MAX phase. The highest products yields were obtained for 10-Nb_2_AlC/TiO_2_ composite reaching 572.0 and 4.4 µmol g^−1^ h^−1^ of CO and H_2_, respectively. This corresponds to approximately 1.72 and 1.2 fold increase in CO and H_2_ generation compared with pristine TiO_2_. This photoactivity enhancement is attributed to the effective interaction between the conductive MAX phase and TiO_2_. Incorporation of Nb_2_AlC into TiO_2_ facilitates migration of electrons, thereby suppressing charge carriers recombination and increasing the availability of electrons for CO_2_ reduction.^68^ The improved charge transfer in the composite material is consistent with the electrochemical impedance results which revealed a lower charge transfer resistance for the composite catalyst as compared to the pure TiO_2_. On further increasing the amount of Nb_2_AlC up to 15% the CO yield deceased to 432 µmol g^−1^ h^−1^ although it remained higher than pure TiO_2_. A slight increase in H_2_ yield was obtained with higher loading of Nb_2_AlC. The reduction in photocatalytic activity at higher loading may be attributed to excessive coverage of the TiO_2_ surface and possible aggregation of Nb_2_AlC particles, which can hinder light absorption and reduce the number of accessible photocatalytic active sites on the surface.^21^ An optimum Nb_2_AlC content is required to achieve a balance between enhanced electron transport and effective utilization of the TiO_2_ surface. In all the cases, CO was obtained as the predominant product, while only a small amount of H_2_ was detected. For 10-Nb_2_AlC/TiO_2_, CO/H_2_ ratio (130) is highest among all the materials which explains the strong selectivity of the composite toward CO formation. Pristine Nb_2_AlC showed very less CO yield (6 µmol g^−1^ h^−1^) and negligible H_2_ which indicates that the MAX phase alone is not the dominant source of product formation under the investigated conditions. This much amount of CO may be originated from the methanol-derived carbon as later verified through the control experiments (Fig. 7d) where CO was observed under N_2_–H_2_O–CH_3_OH atmosphere in the presence of catalyst. This observation indicates that pure Nb_2_AlC is not much effective alone but the prepared Nb_2_AlC/TiO_2_ composites preferentially promote the reduction of CO_2_ to CO and H_2_ generation.

**Fig. 6:**
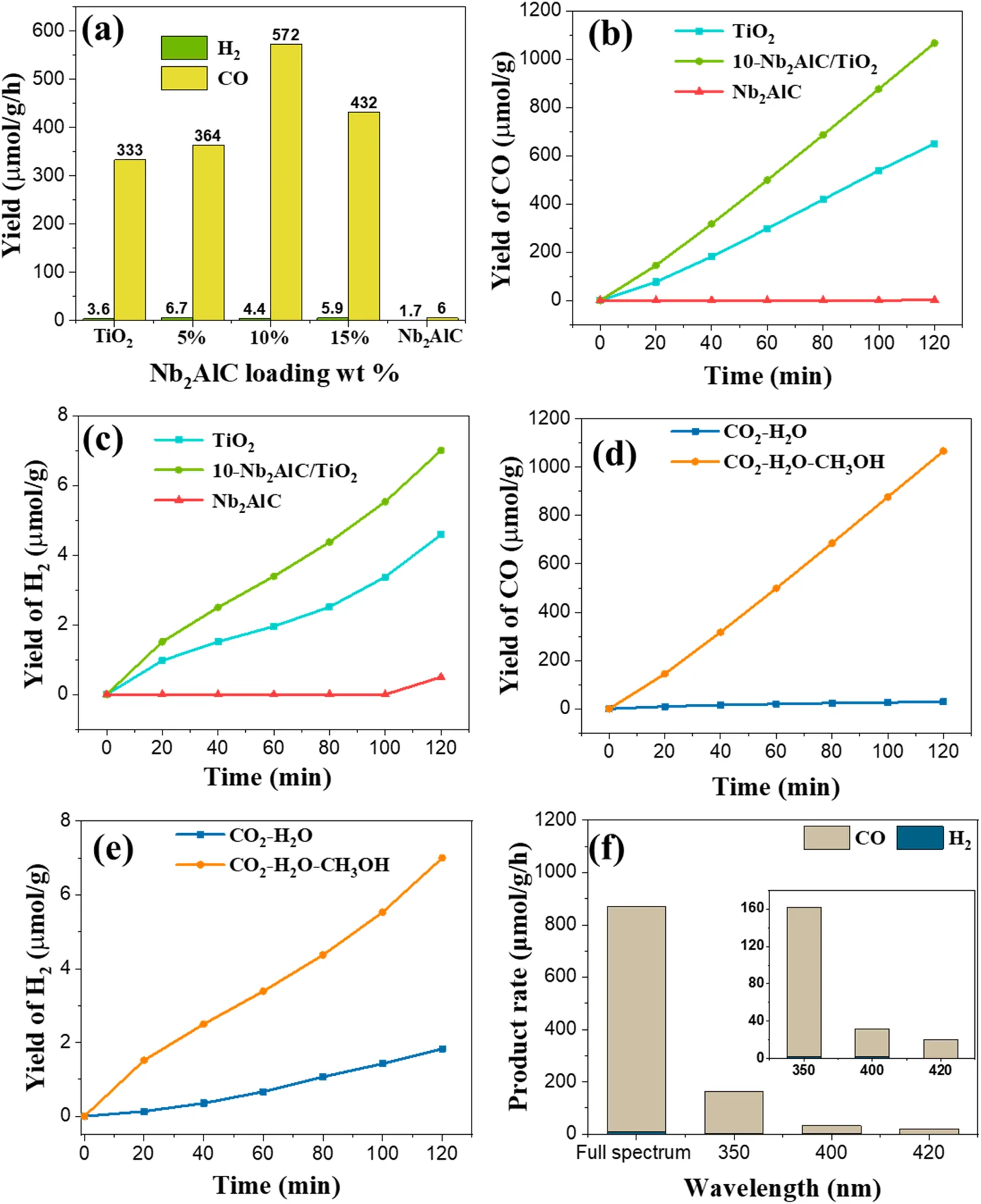
Photocatalytic CO_2_ reduction performance of TiO_2_ and Nb_2_AlC/TiO_2_ composites under different reaction conditions: (a) effect of Nb_2_AlC loadings with feed mixture (CO_2_ + H_2_O + CH_3_OH) at 100 °C (b and c) time dependent evolution of CO and H_2_ over the optimized 10-Nb_2_AlC composite (d and e) influence of methanol as the sacrificial agent on CO and H_2_ evolution, (f) wavelength dependent production rates under full spectrum, BP-350 nm, BP-400 nm, and UVCUT-420 nm optical filters (inset enlarged view) with (CO_2_ + H_2_O + CH_3_OH) feed at 150 °C on the optimized 10-Nb_2_AlC/TiO_2_ composite.

**Fig. 7:**
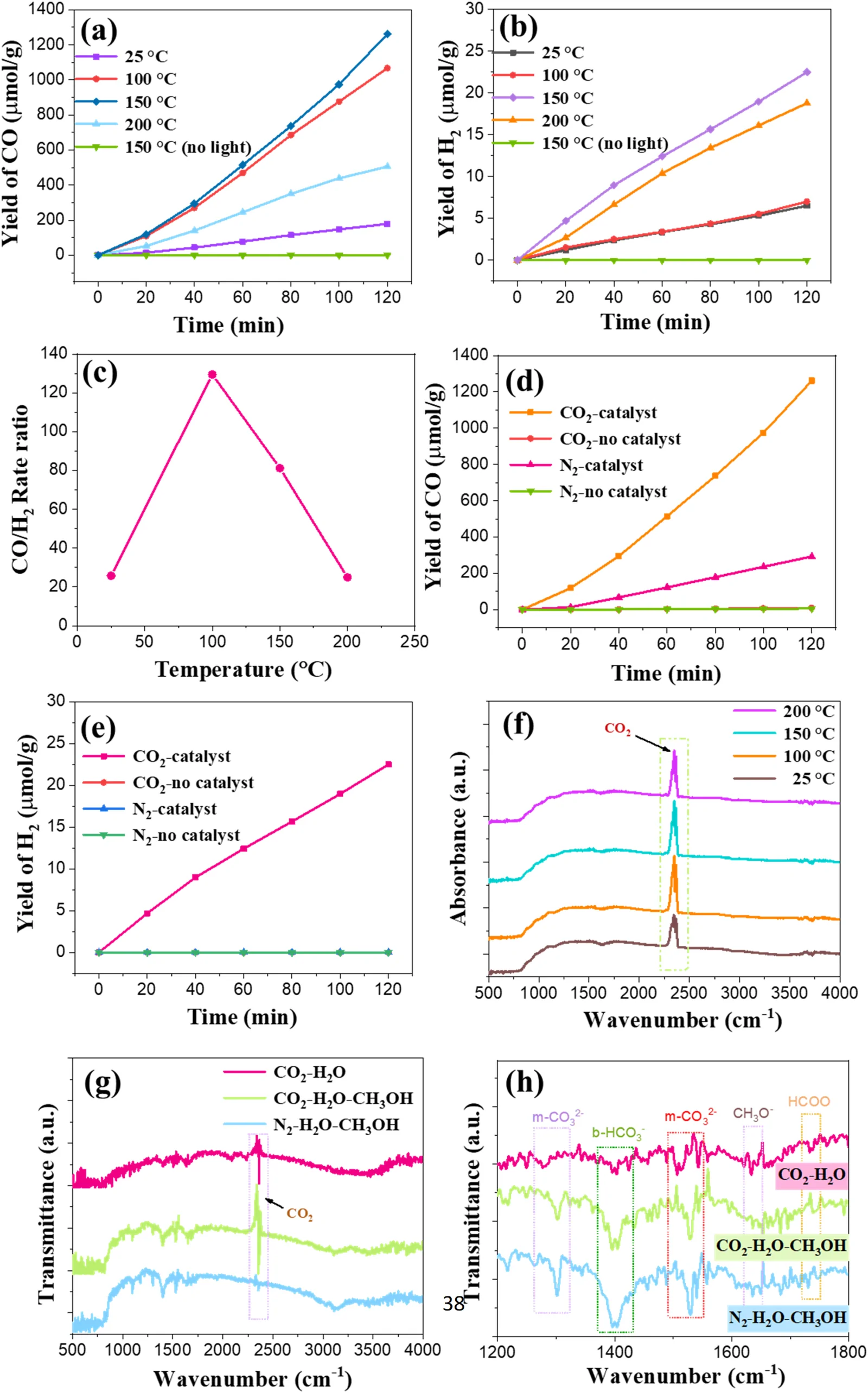
(a and b) Temperature-dependent evolution of CO and H_2_ over 10-Nb_2_AlC/TiO_2_ and (c) variation of the CO/H_2_ ratio with reaction temperature, (d and e) time-dependent CO and H_2_ evolution under CO_2_ and N_2_ atmospheres, with and without the catalyst, in a water–methanol mixture at 150 °C under solar irradiation, (f) *in situ* DRIFT analysis at different reaction temperatures, (g) *in situ* DRIFT analysis under different reaction environments (CO_2_–H_2_O, CO_2_–H_2_O–CH_3_OH, and N_2_–H_2_O–CH_3_OH) at 150 °C, and (h) enlarged 1200–1800 cm^−1^ region showing carbonate, bicarbonate, and oxygenated intermediate-species bands.

Fig. (6b and c) show the time dependence evolution of CO and H_2_ respectively and clearly demonstrate the higher photocatalytic activity of the composite 10-Nb_2_AlC/TiO_2_ over the pure TiO_2_. For both the materials, continuous CO as well as H_2_ evolution is obtained with light illumination. It is clearly observed from the figures that the Nb_2_AlC modified TiO_2_ exhibited significantly higher production yields of both the gaseous products at all the reaction times compared to pure TiO_2_. After 120 min of irradiation, the 10-Nb_2_AlC/TiO_2_ composite achieved a CO yield of approximately 1066 µmol g^−1^, whereas pristine TiO_2_ produced only about 649 µmol g^−1^. The nearly linear increase in CO yield with reaction time indicates stable photocatalytic performance and sustained generation of active charge carriers during the reaction. The enhanced CO formation can be attributed to the effective heterointeraction between Nb_2_AlC and TiO_2_, which promotes the separation and migration of photogenerated electrons and holes. Due to its metallic conductivity, Nb_2_AlC serves as an efficient electron-transfer medium, suppressing charge-carrier recombination and increasing the number of electrons available for CO_2_ reduction. Similar enhancement mechanisms have been reported for Nb-based MXene/TiO_2_ heterostructures, where improved interfacial charge transfer leads to superior photocatalytic activity.^69,70^

Similar to CO production, the amounts of H_2_ also increased gradually with light irradiation (Fig. 6c). After 120 min the composite material exhibited 7.0 µmol g^−1^ of H_2_ yield compared with only 4.5 µmol g^−1^ in pure TiO_2_. The enhancement in H_2_ evolution further confirms the improved utilization of photogenerated charge carriers in the composite system. The amount of H_2_ produced remained always lower than that of CO, indicating that CO_2_ reduction was the dominant reaction pathway under the studied experimental conditions. This preferential formation of CO suggests that the Nb_2_AlC/TiO_2_ heterostructures effectively promote electron transfer toward adsorbed CO_2_ species and high selectivity toward CO formation while suppressing the competing hydrogen evolution reaction. Overall, the time-dependent photocatalytic results reveal that the incorporation of an optimum amount of Nb_2_AlC substantially improves the photoactivity of TiO_2_ for CO_2_ reduction. The enhanced performance is attributed to accelerated charge transport and reduced recombination of charge carriers at the Nb_2_AlC/TiO_2_ composite interface, which facilitate efficient utilization of photogenerated electrons for selective CO production.

#### 3.2.2 Effect of sacrificial agent

Fig. 6d shows the production of CO in absence and presence of methanol in the feed mixture during the photocatalytic reaction. The photocatalytic tests were conducted on the optimized 10-Nb_2_AlC/TiO_2_ composite at 100 °C under solar illumination. In CO_2_–H_2_O feed system without the addition of methanol, CO yield reached only 29 µmol g^−1^ after 120 min of irradiation. On the other hand, in CO_2_–H_2_O–CH_3_OH feed a profound enhancement in CO formation was achieved yielding 1066 µmol g^−1^ which is about 36.75 times higher than with water alone. Similar behavior was also observed for H_2_ production as presented in the Fig. 6e. Highest H_2_ yield up to 7.0 µmol g^−1^ was achieved in the CO_2_–H_2_O–CH_3_OH feed mixture which is about 3.8 times higher than the CO_2_–H_2_O system. These increased results in the presence of methanol can be attributed to its role as a sacrificial agent. During photocatalysis, photogenerated electrons and holes are simultaneously produced on the catalyst surface. Rapid recombination of these charge carriers significantly limits the overall reaction efficiency. Methanol readily reacts with photogenerated holes and hydroxyl radicals, thereby suppressing electron–hole recombination and increase the lifetime of photogenerated electrons.^71^ As a result, a larger number of electrons become available for the reduction of adsorbed CO_2_ and generation of H_2_ molecules. These findings demonstrate the crucial role of sacrificial reagents in improving charge separation and enhancing photocatalytic performance.

Effect of wavelength on the photocatalytic reaction was examined to assess the contribution of TiO_2_ absorption range and the light-absorbing Nb_2_AlC phase to the overall photocatalytic activity. BP350-20K, BP400-20K, and UVCUT-420 nm optical filters were used during the experiments. Fig. 6f shows the highest CO production rates were obtained with full-spectrum radiations (858.8 µmol g^−1^ h^−1^) and progressively decrease was obtained with increase in filter wavelength from 350 to 420 nm. Inserting the 350 nm filter markedly reduced the CO rate to 159 µmol g^−1^ h^−1^ whereas only limited CO was detected under 400 and 420 nm filters (30 and 20 µmol g^−1^ h^−1^, respectively). H_2_ remained as the minor product throughout all the wavelength conditions, confirming the favored CO production over the competing proton-reduction pathway. The regular decline in photoactivity with increase in wavelength is consistent with the TiO_2_ component. Photons transmitted through the 350 nm filter possess sufficient energy to promote electrons from the valence band to the conduction band of TiO_2_. In contrast, irradiation at 400 and 420 nm lies close to or beyond the absorption edge identified from the UV-visible diffuse-reflectance spectrum, resulting in substantially fewer photogenerated charge carriers available for photocatalytic CO_2_ reduction.

#### 3.2.3 Effect of temperature

Temperature-dependent experiments were performed to determine how thermal activation influences the competing CO_2_ reduction and H_2_-evolution reactions. Initially, control experiments were conducted under dark conditions at 150 °C where no detectable products were observed, confirming that the obtained activity originated from a photo-assisted process rather than thermal conversion (Fig. 7a and b). The influence of different reaction temperatures on the photocatalytic performance was studied on the composite 10-Nb_2_AlC/TiO_2_ in the temperature range 25–200 °C with CO_2_–H_2_O–CH_3_OH as the feed mixture. Fig. 7a shows that yield of CO increased continuously with light illumination at all the temperatures but the extent of amount produced varied with temperature as 150 °C > 100 °C > 200°C > 25 °C. At 25 °C, only a limited amount of CO was produced (178 µmol g^−1^), which showed regular increase up to 100 °C (1066 µmol g^−1^). A further rise in temperature up to 150 °C resulted in highest CO generation and reached up to 1261 µmol g^−1^. The enhancement in CO formation at higher temperatures can be attributed to accelerate surface reaction kinetics, improved mobility of reactant molecules, and activation of adsorbed CO_2_ species and more efficient interfacial charge-transfer processes, all of which contribute to improved utilization of photogenerated charge carriers.^72^ Elevated temperatures may also facilitate CO_2_ adsorption and activation on the catalyst surface, thereby promoting the reduction reaction. When the reaction temperature was further increased to 200 °C, a decrease up to 505 µmol g^−1^ in CO yield was observed but still remained higher than that obtained at room temperature. This decrease at high temperature is attributed to the excessive thermal energy that affected the adsorption–desorption equilibrium of CO_2_ and reduced the residence time of adsorbed species on the catalyst surface. Similar influence of temperature has been reported for TiO_2_ where 150 °C to 200 °C is found as the optimal thermodynamic temperature range for CO production.^73^ To further evaluate product selectivity, the CO/H_2_ rate ratio was calculated and plotted as a function of temperature as shown in the Fig. 7c. The ratio increased from 25 °C to a maximum value at 100 °C indicating a strong preference for CO formation. On further increase in temperature to 150 °C and 200 °C, the CO/H_2_ ratio decreased. The decline in CO/H_2_ ratio demonstrates that hydrogen evolution becomes more competitive at elevated temperatures. These results reveal that reaction temperature not only controls the overall photocatalytic activity but also governs the distribution of reduction products. It means moderate temperature favors CO selectivity while excessive heating at higher temperatures promote side reactions and hydrogen evolution, thereby reducing the relative contribution of CO_2_ reduction. Similar temperature-dependent product distributions have been reported for thermally-assisted photocatalytic CO_2_ conversion systems, where temperature influences both adsorption characteristics and interfacial charge-transfer processes.^74^ The results obtained in this study are consistent with several recent reports on carbide-semiconductor systems for light-assisted CO_2_ conversion. Li *et al.* reported selective CO formation through the combined effect of CO_2_ adsorption and conversion at an NH_2_-MXene-MOF interface, demonstrating the importance of interfacial reactant activation in thermally assisted photocatalysis.^46^ Meng *et al.* also showed that a g-C_3_N_4_/TiO_2_/MXene heterojunction could promote low-temperature CO_2_ to CO conversion under photothermal conditions, which was attributed to the combined contribution of light absorption and interfacial charge transfer.^47^ Similarly, nitrogen-doped TiO_2_/Ti_3_C_2_ MXene composites have been reported to improve photocatalytic CO_2_ reduction because of enhanced CO_2_ adsorption and more effective separation of photogenerated charge carriers.^48^ Other studies using Nb_2_C-supported Cu sites and Mo_2_C/TiO_2_ have further shown that transition-metal carbides can modify the electronic environment of semiconductor or metal active sites and improve CO_2_ reduction performance.^50,51^ In agreement with these reports, the higher CO production observed for Nb_2_AlC/TiO_2_ compared with pristine TiO_2_ may result from the combined effects of improved electron transport, interfacial CO_2_ activation, and temperature-assisted reaction kinetics. The present results therefore support Nb_2_AlC/TiO_2_ heterostructure ability to tune syngas composition through temperature regulation, which may be advantageous for the controlled production of carbon-based fuels and chemical feedstocks. Overall, Nb_2_AlC/TiO_2_ heterojunction promotes charge separation and enables selective CO_2_ to CO photo reduction, while reaction temperature controls CO/H_2_ syngas composition. Photocatalytic performance of TiO_2_ and optimized 10-Nb_2_AlC/TiO_2_ w.r.t. production rate, selectivity (%), and CO/H_2_ ratio under different conditions is shown in the Table 1. Also, a comparative summary with existing TiO_2_- based composites reported in CO_2_ photoreduction is presented in Table 2. To further evaluate the photon utilization efficiency of the prepared photocatalysts, the apparent quantum efficiency (AQE) was estimated using eqn (1) and (2) under the applied irradiation conditions. The calculated values of (AQE %) for both the products are shown in the Table 1. The AQE for CO increased from 0.1% at 25 °C to 0.7% at 100 °C, reaching a maximum of 1.0% at 150 °C, followed by a decrease to 0.2% at 200 °C. In comparison, pristine TiO_2_ exhibited AQE of approximately 0.4% at 100 °C. The AQE for H_2_ remained substantially lower, approximately 0.01% under the investigated optimum condition. The maximum apparent AQE observed at 150 °C is consistent with the highest CO and H_2_ production rates obtained at this temperature, suggesting that the enhanced catalytic performance at 150 °C is accompanied by more effective utilization of the incident light. The decrease in AQE at 200 °C further indicates that simply increasing the reaction temperature does not continuously improve photon utilization, consistent with the observed temperature-dependent catalytic behavior.

**Table 1:** Performance of photocatalysts w.r.t. production rate, apparent quantum efficiency, selectivity (%), and CO/H_2_ ratio under different conditions^a^

Photocatalyst	Feed and temp.	Production rate (µmol g^−1^ h^−1^)		AQE (%)		Selectivity (%)		CO/H_2_ ratio
		CO	H_2_	CO	H_2_	CO	H_2_	
TiO_2_	CO_2_ + H_2_O + CH_3_OH (100 °C)	332.8	3.6	0.4	—	98.93	1.07	92.4
10-Nb_2_AlC/TiO_2_	CO_2_ + H_2_O + CH_3_OH (25 °C)	92.8	3.6	0.1	—	96.27	3.73	25.7
10-Nb_2_AlC/TiO_2_	CO_2_ + H_2_O + CH_3_OH (100 °C)	572.0	4.4	0.7	—	99.24	0.76	130
10-Nb_2_AlC/TiO_2_	CO_2_ + H_2_O + CH_3_OH (150 °C)	858.8	10.5	1.0	0.01	98.79	1.21	81.7
10-Nb_2_AlC/TiO_2_	CO_2_ + H_2_O + CH_3_OH (200 °C)	200.2	8.0	0.2	0.01	96.16	3.84	25.0

a AQE values were estimated using an assumed incident irradiance of 1 sun (100 mW cm^−2^), illuminated area of 12.56 cm^2^, and effective wavelength of 420 nm; so, the values represent apparent or estimated AQE not the calibrated values.

**Table 2:** Comparative summary with existing TiO_2_- based composites reported in CO_2_ photoreduction^a^

Catalyst	Gaseous feed	Parameters	Production rate (µmol g^−1^ h^−1^)	Reference
10-Nb_2_AlC/TiO_2_	CO_2_–H_2_O–CH_3_OH	Continuous fixed bed reactor, 100 mW cm^−2^	CO = 858.8	Present study
			H_2_ = 10.5	
10% V_2_AlC/TiO_2_	CO_2_–H_2_O	Fixed bed photoreactor	CO = 522	80
			CH_4_ = 78.3	
Ti_3_AlC_2_/TiO_2_	CO_2_–CH_4_–H_2_O	Fixed-bed	CO = 930 H_2_ = 188	81
	CO_2_–CH_4_–H_2_O	Monolith	CO = 575H_2_ = 660	
		150 mW cm^−2^ lamp		
Nb_2_C/TiO_2_	CO_2_–H_2_O	300 W Xe lamp	CH_4_ = 7.2	45
TiO_2_/CsPbBr_3_	CO_2_–H_2_O, TEOA	300 W Xe lamp	CO = 625	82
C/Ag@urchin-like TiO_2_	CO_2_, H_2_O	300 W Xe lamp	CH_4_ = 5.46	83
			CO = 1.51	
TiO_2_@Bi_2_MoO_6_	CO_2_, H_2_O	300 W Xe lamp	CO = 30.67	84
Cu_2_O/Mo_2_C/TiO_2_	CO_2_, 0.5 M KHCO_3_	LED light, *E* = 5 mW cm^−2^	CH_3_OH = 36.3	85

a The reported studies are provided only for comparative summary. This table do not directly claim any material as the best and superior than the others. Order in performance is not valid because the cited studies differ in light source, irradiation wavelength, photon flux, reactor geometry, cocatalyst composition.

To examine the contribution of methanol in CO formation and dependence on the reaction atmosphere and catalyst, control experiments were conducted at 150 °C under solar irradiation using a water–methanol mixture (Fig. 7d and e). In the presence of 10-Nb_2_AlC/TiO_2_, the CO yield under CO_2_ reached 1261 µmol g^−1^ after 120 min, whereas replacing CO_2_ with N_2_ reduced the yield to approximately 292 µmol g^−1^. CO production under CO_2_ was approximately 4.3 times that under N_2_. This CO formation detected under N_2_ environment is attributed to the methanol conversion under the applied light and temperature conditions, in the presence of catalyst, while the additional CO formed under CO_2_ supports a substantial contribution from CO_2_ conversion. The catalyst-free experiments showed negligible CO amounts under either atmosphere, supporting a substantial catalytic contribution to CO formation. These results support the assignment of methanol mainly as a sacrificial hole-scavenger, but also its simultaneous participation in photo-oxidation or reforming reactions to some extent. H_2_ production reached approximately 22.5 µmol g^−1^ under CO_2_ in the presence of the catalyst, while the remaining conditions with N_2_ and without catalyst showed negligible H_2_. These results demonstrate that the highest product yields were obtained with both the catalyst and a CO_2_ atmosphere under the investigated conditions and supporting a substantial catalytic contribution to CO_2_ reduction. No CH_4_ or other carbon-containing gaseous products were detected by the online gas chromatograph. It can be attributed to the continuous-flow reactor where minor products produced may flow out from the reaction chamber continuously or have remained below the detection limit.

To further investigate the effect of temperature on the interactions of CO_2_ with the photocatalyst, *In situ* DRIFT spectra were taken as presented in the Fig. 7f. The band centered at round 2340 cm^−1^ is assigned predominantly to the asymmetric stretching vibration of molecular CO_2_.^75^ It can be seen that at 25 °C the peak is less intense while becomes more pronounced upon increasing the temperature from 25 to 100 °C and remains of highest intensity at 150 °C, confirming that the CO_2_ concentration at the surface of the catalyst increases with temperature. These results are in well agreement with the highest product yield obtained for the photocatalytic experiments at 150 °C. At this temperature, maximum number of CO_2_-derived species undergo conversion and generating the maximum amount of CO. At higher temperature of 200 °C, slightly less intense peak is observed which indicates shorter residence time of CO_2_-derived species, and thermal desorption of CO_2_ molecules that ultimately lead to decreased CO.

To distinguish the individual contributions of CO_2_ and methanol, and detailed insight into the surface intermediates formed during the reaction, *in situ* DRIFT measurements were performed under three different reaction environments including CO_2_–H_2_O, CO_2_–H_2_O–CH_3_OH, and N_2_–H_2_O–CH_3_OH at the optimum temperature of 150 °C during irradiation. The complete spectra are shown in Fig. 7g, while the region associated with the reaction intermediates (1200–1800 cm^−1^) is enlarged in Fig. 7h. A distinct band near 2340 cm^−1^ is observed in the CO_2_-containing feeds and is assigned to the asymmetric stretching vibration of adsorbed molecular CO_2_. This band is not much dominant in the N_2_–H_2_O–CH_3_OH spectrum, but the low intensity appearance can be attributed to either atmospheric CO_2_ or from the decomposition of methanol at the given reaction temperature and light conditions. It is important to note that the CO_2_–H_2_O spectrum also shows weak features in the intermediate region (1200–1800 cm^−1^), indicating that carbonate, bicarbonate, and oxygenated-surface species can even form in the absence of methanol and indicate contribution directly from CO_2_. These observations demonstrate that methanol is not essential for the initial adsorption and activation of CO_2_ on the catalyst surface. However, the corresponding bands become more pronounced under the methanol addition, suggesting that methanol modifies the surface reaction environment and promotes the accumulation or transformation of oxygen-containing intermediates. The bands located ∼ in the 1380–1400 cm^−1^ and 1500–1520 cm^−1^ regions can be associated with surface carbonate species (CO_3_^2−^), whereas the feature around 1450–1470 cm^−1^ is attributed to bicarbonate (HCO_3_^−^) species.^76,77^ Additional bands appearing near 1655 and 1740 cm^−1^ indicate the formation of oxygenated methoxy/formate species (CH_3_O^−^ and HCOO^−^) during the reaction.^70,78^ This analysis indicates that CO_2_ can adsorb and form carbonate/bicarbonate/oxygenated intermediate species even without methanol. The combined feed (CO_2_–H_2_O–CH_3_OH) produces the more intense intermediate spectrum, supporting a coupled reaction pathway in which CO_2_ adsorption and activation occur alongside methanol-assisted charge-carrier consumption and surface oxidation reactions.

Similar to CO production, H_2_ also increased continuously with irradiation time (Fig. 7b) but followed different trend with temperature observed as 150 °C > 200 °C > 100 °C ∼25 °C. The increase in hydrogen evolution with temperature can be attributed to enhanced proton reduction kinetics and accelerated surface reactions involving photogenerated electrons. The relatively high H_2_ production at 150 °C indicates that elevated temperatures favor the hydrogen evolution pathway more strongly than CO_2_ reduction. Similar results have been reported for Pt/TiO_2_ where no hydrogen was detected below 250 °C, but at elevated temperatures up to 600 °C H_2_ generation promoted to significant amounts.^79^ These results support that water dissociation is thermally prompted at elevated temperatures and favors H_2_ over CO production.

#### 3.2.4 Cyclic stability

The stability and reusability tests were conducted on 10-Nb_2_AlC/TiO_2_ composite with CO_2_–H_2_O–CH_3_OH as the feed mixture at 100 °C. After every completed experiment, lamp was turned off and reactor was completely cleaned with continuous flow of feed gas until no gaseous products were found inside the reactor left from the previous experimental run. Fig. (8a and 8b) presents the products yield for CO and H_2_ respectively, for five consecutive photocatalytic cycles. As clearly seen from the Fig. 8a, CO generation was sustained throughout all cycles, indicating that the catalyst remained photocatalytically active after repeated use. Interestingly, the CO yield increased after the first cycle and remained higher in the subsequent runs. This behavior suggests that the catalyst surface may undergo *in situ* activation during the initial photocatalytic reaction, possibly through removal of weakly adsorbed impurities, improved adsorption of CO_2_, and exposure of additional active sites on the surface of the composite under reaction conditions. The higher CO yield observed after the first cycle indicates an activation stage, while the sustained CO production over five cycles confirms the durability of the Nb_2_AlC/TiO_2_ heterostructures. A similar trend was also observed for H_2_ evolution as shown in Fig. 8b, where the yield increased markedly in the second cycle and then showed a moderate decrease in later cycles. As H_2_ was produced as a minor product and showed some variation during repeated cycles, which may be due to its low concentration produced and the competitive nature of hydrogen evolution with CO formation. The continuous formation of H_2_ and remaining detectable throughout the recycling experiments confirms the photoactivity of the composite catalyst. The Nb_2_AlC/TiO_2_ catalyst showed excellent stability for CO production over five cycles, while H_2_ remained a minor product with moderate cycle-to-cycle variation.

**Fig. 8:**
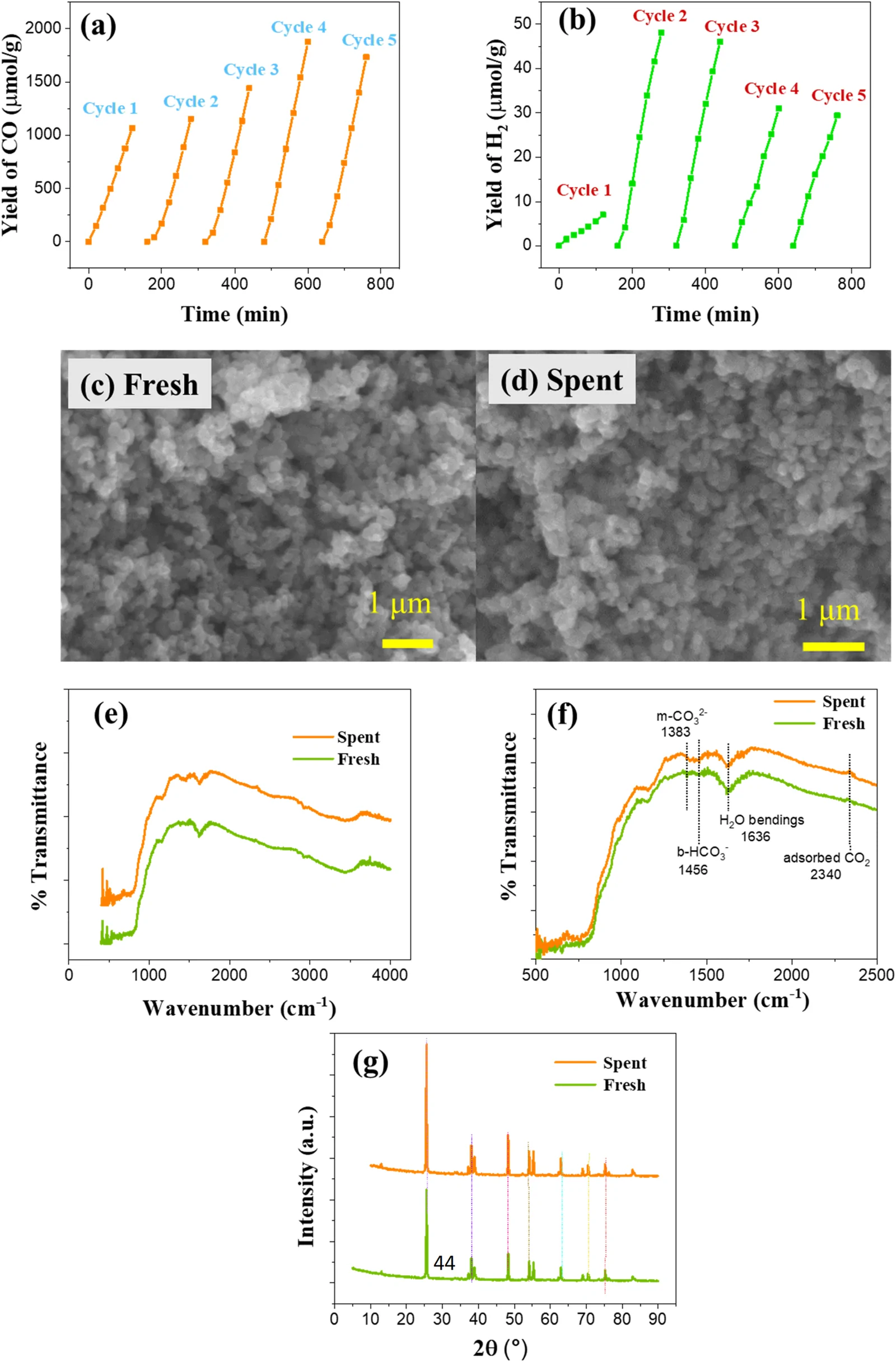
Stability and recyclability assessment of the optimized 10-Nb_2_AlC/TiO_2_ composite: (a and b) evolution of CO and H_2_ with time in five consecutive photocatalytic cycles, SEM micrographs of the (c) fresh and (d) spent composite after reaction, (e and f) FTIR with enlarged view, and (g) XRD of fresh and spent photocatalyst after reaction.

Further, the chemical structure and morphology of the composite are also maintained after repeated reaction cycles. The spent catalyst was recovered and examined for morphological and structural similarity with the fresh catalyst. Fig. (8c and 8d) show the SEM images of the fresh and spent catalyst where no significant change is observed after the photocatalytic reaction. Similarly, FTIR spectra of fresh and spent material are shown in the Fig. 8e, where no additional peaks were observed and both the fresh and spent material exhibited similar peak positions. The enlarged spectrum shown in Fig. 8f revealed weak bands at 1383 and 1456 cm^−1^ in the spent material, did not appered in the fresh sample. This indicates the surface adsorbed carbonate (CO_3_^2−^) and bicarbonate (HCO_3_^−^) species, respectively in the material during the reaction.^76–78^ The peak observed at 1636 cm^−1^ corresponding to the bending mode of adsorbed H_2_O molecules becomes less intense in the spent material as compared to the fresh one.^86^ This is attributed to the removal of moisture from the material after repeated heat treatment during the reaction cycles. A slight increase in the peak intensity at 2340 cm^−1^ is seen indicating more number of CO_2_ molecules adsorbed in the spent sample left after reaction.^75^ A little peak seen in the fresh sample is due to the presence of atmospheric CO_2_ molecules. Both the analysis confirmed the structural stability of the Nb_2_AlC/TiO_2_ heterostructures after five cycles of photocatalytic process. The XRD patterns of the fresh and spent catalysts after five reaction cycles exhibit the same characteristic diffraction peaks, with no additional reflections attributable to Nb_2_O_5_ or other crystalline oxidation products (Fig. 8g). The increase in peak intensity for the spent catalyst may result from removal of weakly adsorbed species, differences in sample packing, or improved crystallinity during repeated thermal treatment. Importantly, no phase collapse or detectable crystalline-phase transformation was observed after the catalytic cycles. These XRD results, together with the SEM and FTIR analyses, indicate that the main crystalline structure of the catalyst was retained after reuse.

### 3.3 Mechanism

Fig. 9 shows the photothermal catalytic reduction of CO_2_ over Nb_2_AlC/TiO_2_ heterostructures proposed on the basis of the optical, electrochemical, and photocatalytic results. Upon light illumination, TiO_2_ absorbs photons with energy equal to or greater than its band gap, resulting in the generation of electron–hole pairs (eqn (3)). In pristine TiO_2_, a considerable fraction of the photogenerated charge carriers undergoes rapid recombination, limiting the number of electrons available for reduction reactions. After incorporation of Nb_2_AlC, the photogenerated electrons are readily transferred from the conduction band of TiO_2_ to the conductive Nb_2_AlC MAX phase (eqn (4)) because of its metallic conductivity and favorable electron-transport characteristics. The optical and electrochemical results suggest that Nb_2_AlC performs complementary functions in the Nb_2_AlC/TiO_2_ composite. The UV-vis DRS spectrum of Nb_2_AlC shows broad absorption across the investigated wavelength range, and the composite exhibits stronger absorption than pristine TiO_2_. This behavior indicates that Nb_2_AlC can contribute to light harvesting and photothermal activation. In parallel, the lower charge-transfer resistance observed in EIS, together with the enhanced CV response and the Mott–Schottky results, indicates improved charge transport after Nb_2_AlC incorporation. Therefore, Nb_2_AlC is considered both a broadband light-absorbing component and a conductive electron-transfer medium. The transferred electrons accumulate on the Nb_2_AlC surface and participate in the reduction of adsorbed CO_2_ molecules. Methanol and water simultaneously consume the photogenerated holes that remain in the valence band of TiO_2_, producing protons through the reactions represented in eqn (5) and(6). By acting as a sacrificial hole-scavenger, methanol continuously removes holes from the TiO_2_ surface, thereby suppressing charge-carrier recombination and prolonging the availability of photogenerated electrons. As a result, a greater number of electrons can participate in the reduction of CO_2_. This behavior is consistent with the markedly higher CO evolution observed for the CO_2_–H_2_O–methanol system compared with the CO_2_–H_2_O system. The interaction of methanol with water provides an additional source of electrons and protons, as described in eqn (7). The electrons transferred to the Nb_2_AlC surface are subsequently utilized for the reduction of adsorbed CO_2_ molecules. According to eqn (8), the conversion of CO_2_ into CO proceeds through a two-electron/two-proton reduction pathway. At the same time, a small proportion of these electrons contributes to the hydrogen evolution reaction, where two protons combine with two electrons to form H_2_ (eqn (9)). Although hydrogen is generated as a parallel product, its amount remains much lower than that of CO, indicating that electron utilization is predominantly directed toward CO_2_ reduction in the Nb_2_AlC/TiO_2_ heterostructure rather than proton reduction. The catalytic results further demonstrate that reaction temperature plays an important role in determining both catalytic activity and product selectivity. Increasing the temperature from 25 to 150 °C enhanced CO formation, which can be attributed to faster surface reaction kinetics, improved mobility of reactant molecules, and more effective activation of adsorbed CO_2_ species. At 200 °C, however, the CO yield decreased, probably because excessive heating weakened the adsorption of CO_2_-derived intermediates and reduced their residence time on the catalyst surface. In contrast, the relative contribution of H_2_ increased at elevated temperature, suggesting that proton reduction and water/methanol-related hydrogen formation became more competitive. The decrease in the CO/H_2_ ratio at higher temperature therefore reflects a change in product distribution rather than a loss of photocatalytic activity. The superior performance of the 10-Nb_2_AlC/TiO_2_ photocatalyst arises from the combined influence of rapid electron transport across the heterostructure, efficient suppression of charge recombination, continuous removal of holes by methanol, and the beneficial effect of moderate photothermal heating on the surface reaction kinetics. The proposed reaction pathway is summarized in eqn (3)–(9).

3 TiO_2_ + *hv* → TiO_2_ (e_CB_^−^) + TiO_2_ (h_VB_^+^)

4 TiO_2_ (e_CB_^−^) + Nb_2_AlC → Nb_2_AlC(e^−^) + TiO_2_

5 H_2_O + h^+^ → H^+^ + ˙OH

6 CH_3_OH + h^+^ → ˙CH_2_OH + H^+^

7 CH_3_OH + H_2_O → CO_2_ + 6H^+^ + 6e^−^

8 CO_2_ + 2H^+^ + 2e^−^ → CO + 2H_2_O

9 2H^+^ + 2e^−^ → H_2_

**Fig. 9:**
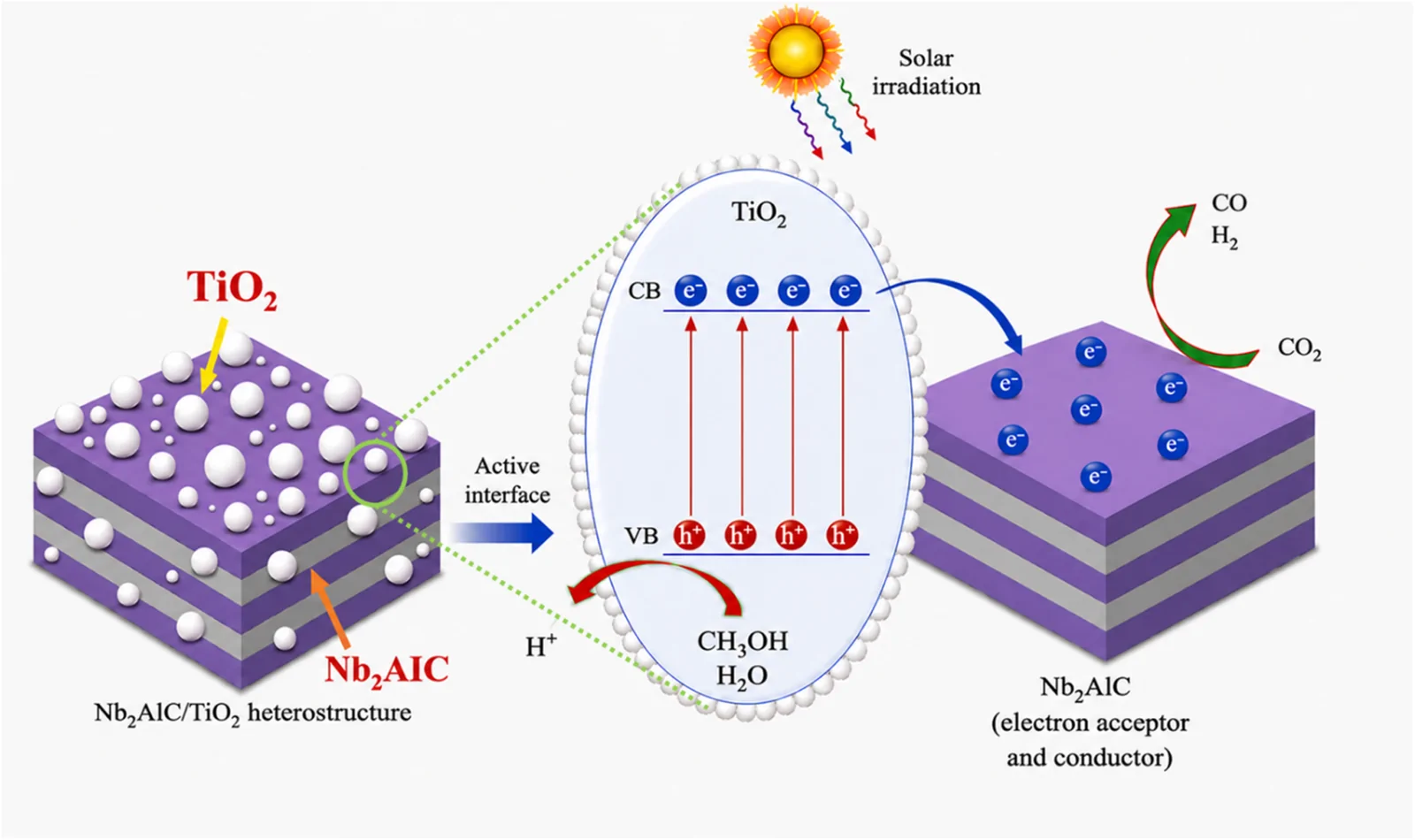
Schematic illustration of the charge transfer mechanism in Nb_2_AlC/TiO_2_ heterostructures in CO_2_ photoreduction.

## 4. Conclusion

In conclusion, a conductive Nb_2_AlC MAX phase was successfully integrated with TiO_2_ through a simple ultrasonication-assisted approach to construct Nb_2_AlC/TiO_2_ heterostructures for photothermal CO_2_ reduction under solar irradiation. Among the investigated compositions, the 10% Nb_2_AlC/TiO_2_ composite exhibited outstanding catalytic performance, achieving maximum evolution rates of 858.8 and 10.5 µmol g^−1^ h^−1^ of CO and H_2_ respectively, with water–methanol mixture at 150 °C. The key novelty of this study lies in demonstrating Nb_2_AlC MAX phase as a conductive and photothermally active component for TiO_2_ in continuous-flow CO_2_ conversion. Unlike usual approaches based on etched MXenes, this system combines the structural stability of the Nb_2_AlC phase with the photocatalytic functionality of TiO_2_, while temperature and irradiation control provide an additional means of regulating product formation. The enhanced activity is associated with the synergistic interaction between conductive Nb_2_AlC phase and TiO_2_, which promoted increased light absorption, rapid interfacial electron transfer, suppressed charge-carrier recombination, and prolonged the lifetime of photogenerated electrons available for CO_2_ reduction. XRD, FTIR, UV-vis DRS, PL, XPS, EIS, and CV measurements collectively demonstrating the successful formation of an efficient heterostructure with improved charge-transport characteristics. The photothermal experiments also showed that reaction temperature strongly influenced both catalytic activity and relative distribution of the products. This behavior was further supported by *In situ* DRIFT measurements which showed strongest CO_2_ response at 150 °C consistent with maximum CO production at this temperature. Also, progressive evolution of carbonate, bicarbonate and other CO_2_-derived surface species with time supported CO_2_ activation and transformation on the catalyst surface. Moderate temperatures enhanced CO_2_ activation and subsequent reaction occurring on the catalyst surface reaction kinetics, whereas further temperature elevation increasingly favored hydrogen evolution, resulting in a gradual change in the CO/H_2_ product ratio. This temperature-dependent behavior demonstrate that the composition of generated syngas can be adjusted by simply controlling the reaction temperature without altering catalyst composition. CO formation was strongly promoted in the presence of CO_2_, although the control experiments indicate that methanol-derived carbon may also contribute to a fraction of the detected CO. Methanol acted as an efficient hole-scavenger, substantially suppressing the charge recombination and allowing a large fraction of electrons to participate in CO_2_ reduction. Reusability tests confirmed that catalyst also maintained both its crystal structure and photocatalytic performance over successive reaction cycles, demonstrating excellent stability under photothermal operating conditions. These findings establish Nb_2_AlC/TiO_2_ as a promising platform for controllable photothermal CO_2_ reduction.

## Conflicts of interest

There are no conflicts to declare.

## Abbreviation

AQE: Apparent quantum efficiency
MAX: MAX phase
XRD: X-ray diffraction
FTIR: Fourier-transform infrared spectroscopy
DRIFT: Diffuse reflectance infrared Fourier-transform spectroscopy
SEM: Scanning electron microscopy
UVDRS: Ultraviolet-visible diffuse reflectance spectroscopy
PL: Photoluminescence
EIS: Electrochemical impedance spectroscopy
CV: Cyclic voltammetry
XPS: X-ray photoelectron spectroscopy
Symbol: Meaning unit
*λ*
: Wavelength of incident light nm
*h*
: Planck's constant J s
*c*
: Speed of light ms^−1^
*R*
: Product evolution rate µmol g^−1^ h^−1^
*m*_cat_
: Catalyst mass g
*N*_photon_
: Incident photon flux µmol photons per h
*T*
: Reaction temperature °C
*t*
: Irradiation time min
AQE: Apparent quantum efficiency %

## Supplementary Material

RA-OLF-D6RA09084B-s001

## Data Availability

The data supporting the findings of this study are already provided in the manuscript.

## References

[cit1] Alli Y. A., Bamisaye A., Bamidele M. O., Etafo N. O., Chkirida S., Lawal A., Hammed V. O., Akinfenwa A. S., Hanson E., Nwakile C., Kazeem K. O., Ayanwunmi R. J., Ige A. S., Parga Torres J. R., Al Nageim H. (2024). Transforming waste to wealth: Harnessing carbon dioxide for sustainable solutions. Results Surf. Interfaces.

[cit2] Alli Y. A., Ejeromedoghene O., Dembaremba T. O., Adawi A., Alimi O. A., Njei T., Bamisaye A., Kofi A., Anene U. Q., Adewale A. M., Yaqub Z. T., Oladele M. E., Jimoh L., Oni S. O., Ogunlaja A. S., Xu B. B. (2025). Perspectives on the status and future of sustainable CO_2_ conversion processes and their implementation. Carbon Capture Sci. Technol..

[cit3] Nunes L. J. R. (2023). The Rising Threat of Atmospheric CO_2_: A Review on the Causes, Impacts, and Mitigation Strategies. Environments.

[cit4] Yergaziyeva G., Kuspanov Z., Mambetova M., Khudaibergenov N., Makayeva N., Daulbayev C. (2024). Advancements in catalytic, photocatalytic, and electrocatalytic CO_2_ conversion processes: Current trends and future outlook. J. CO_2_ Util..

[cit5] Fu C., Wan Z., Yang X., Zhang J., Zhang Z. (2024). Artificial CO_2_ photoreduction: a review of photocatalyst design and product selectivity regulation. J. Mater. Chem. A.

[cit6] Islam A., Malek A., Teo S. H., Marwani H. M., Rahman M. M., Asiri A. M., Khan M. A. R., Taufiq-Yap Y. H., Awual M. R. (2023). Smart materials for CO_2_ conversion into renewable fuels and emission reduction. Sustainable Mater. Technol..

[cit7] Zhang F., Li Y.-H., Qi M.-Y., Yamada Y. M. A., Anpo M., Tang Z.-R., Xu Y.-J. (2021). Photothermal catalytic CO_2_ reduction over nanomaterials. Chem Catal..

[cit8] Zhang J., Chen H., Duan X., Sun H., Wang S. (2023). Photothermal catalysis: From fundamentals to practical applications. Appl. Mater. Today.

[cit9] Tai J. Z., Alias H., Shamjuddin A., Yusof M. S. M., Fan W. K., Tahir M. (2026). Solar-Driven CO_2_ Hydrogenation to Renewable Products: A Review of Strategies for Selectivity Tuning. Energy Fuels.

[cit10] Dong Y., Song R., Zhang Z., Han X., Wang B., Tao S., Zhao J., Alodhayb A. N., Chen Z., Yi X. (2024). Advances in photothermal CO_2_ hydrogenation catalysis for C1 molecules. Cell Rep. Phys. Sci..

[cit11] Tang Y., Li Y., Bao W., Yan W., Zhang J., Huang Y., Li H., Wang Z., Liu M., Yu F. (2023). Enhanced dry reforming of CO_2_ and CH_4_ on photothermal catalyst Ru/SrTiO_3_. Appl. Catal., B.

[cit12] Li Q., Lu C., Karin J. D., Zhang Y., Ling C., Zhang X., Zhou Z., Wang J., Prezhdo O. V. (2025). Dual-Defect Donor–Acceptor Pairing in Metal Oxide Semiconductors for Enhanced CO_2_ Photoreduction. Nano Lett..

[cit13] Ge X., Yuan L., Sun Q., Lin T., Chen W., Sun J., Yu H., Jin J., Liu F., He H. (2026). Minute-level alkylation of heteroarenes via a photo-Fenton mechanism in continuous flow. Green Chem..

[cit14] Gede M., Ignacz G., De Castro C. S., Dereli B., Ferree M., Laquai F., Cavallo L., Szekely G. (2026). Tailoring polymers of intrinsic microporosity as photoredox catalysts for continuous-flow reaction–separation processes. Nat. Commun..

[cit15] Azmoon P., Farhadian M., Pendashteh A. (2025). Design and fabrication of continuous flow fixed-bed photoreactor to photocatalytic degradation of oil-well produced water using conductive photocatalytic fibers. Chem. Eng. J..

[cit16] Dasireddy V. D. B. C., Vengust D., Likozar B., Kovač J., Mrzel A. (2021). Production of syngas by CO_2_ reduction through Reverse Water–Gas Shift (RWGS) over catalytically-active molybdenum-based carbide, nitride and composite nanowires. Renew. Energy..

[cit17] Machado M., de Souza Junior R. L., de Almeida J. M. A. R., Romano P. N., Garcia M. A. S. (2024). Syngas enhancement for Fischer-Tropsch integration via solid oxide electrolyzer cell co-electrolysis with or without methane. IScience.

[cit18] Chen B., Feng M., Chen Y., Yang J., Liu Y. (2024). Performing electrocatalytic CO_2_ reduction reactions at a high pressure. Carbon Neutralization.

[cit19] Li Y., Pei X., Wang Z.-j., Shi L., Song H., Ye J. (2024). Photothermal Catalytic CO_2_ Conversion to Value-Added Chemicals: Progress and Prospects. ACS Sustain. Chem. Eng..

[cit20] Lee H. S., Choi J., Lee J. Y., An J. E., Vu T. H., Bui V. D., Kamyab H., Kim H. H., Aminabhavi T. M., Vasseghian Y. (2025). Photocatalytic CO_2_ conversions on copper nanoparticles investigated by Raman spectral changes using convolutional neural networks. Sustainable Mater. Technol..

[cit21] Liu L., Behmadi R., Tan R., Rahimi H., Shoja Razavi R., Liu Y., Kamyab H., Gnanasekaran L., Hojjati-Najafabadi A., He Y. (2026). CO_2_ conversion into fuels and value-added chemicals: from molten salt catalysts to metal–organic and covalent–organic frameworks. J. Mater. Sci..

[cit22] Sharma S., Kumar N., Mari B., Chauhan N. S., Mittal A., Maken S., Kumari K. (2021). Solution combustion synthesized TiO_2_/Bi_2_O_3_/CuO nano-composites and their photocatalytic activity using visible LEDs assisted photoreactor. Inorg. Chem. Commun..

[cit23] Yuan Z., Zhu X., Gao X., An C., Wang Z., Zuo C., Dionysiou D. D., He H., Jiang Z. (2024). Enhancing photocatalytic CO_2_ reduction with TiO_2_-based materials: Strategies, mechanisms, challenges, and perspectives. Environ. Sci. Ecotechnol..

[cit24] Sharma S., Mittal A., Chauhan N. S., Saini S., Yadav J., Kushwaha M., Chakraborty R., Sengupta S., Kumari K., Kumar N. (2022). Mechanistic investigation of RhB photodegradation under low power visible LEDs using a Pd-modified TiO_2_/Bi_2_O_3_ photocatalyst: Experimental and DFT studies. J. Phys. Chem. Solids.

[cit25] Chen J., Li T., An X., Fu D. (2024). Photocatalytic Reduction of CO_2_ by TiO_2_ Modified Materials: Recent Advances and Outlook. Energy Fuels.

[cit26] Li Y., Wei Y., He W., Tang Z., Xiong J., Zhao Z. (2023). Ordered macroporous structured TiO_2_-based photocatalysts for CO_2_ reduction: a review. Chin. Chem. Lett..

[cit27] Chen X., Mao S. S. (2007). Titanium dioxide nanomaterials: synthesis, properties, modifications, and applications. Chem. Rev..

[cit28] Kamyab H., Khademi T., Rezania S., Rajendran S., Shanmugapriya D., Yuzir A., Chelliapan S., Shekofteh-Gohari M. (2026). S-scheme Heterojunction Engineering of ZnO/ZnIn_2_S_4_ for Efficient Solar-Driven Oxygen Evolution in Photoelectrochemical Water Splitting. J. Solid State Chem..

[cit29] Kamyab H., Khademi T., Chelliapan S., Yusuf M., Rajendran S., Shekofteh-Gohari M. (2025). Enhancing RhB photocatalytic degradation with ZnO/Sb_2_MoO_6_ Z-scheme photocatalyst: Evaluation of performance and mechanism. Mater. Res. Bull..

[cit30] Shooshtari F. K., Mohtaram M. S., Roohparvarzadeh P., Zerafat M. M., Kamyab H., Moradi H., Rajendran S., Sabbaghi S. (2025). Efficient charge transfer in rheum ribes waste-derived biochar-supported Bi_2_MoO_6_ nanocomposites for visible-light-driven photocatalytic degradation of antibiotics. J. Water Process Eng..

[cit31] Kamyab H., Khademi T., Chelliapan S., Yuzir A., Rocchio D., Khorami M., Shanmugapriya D. (2026). Adsorptive Removal of Simazine Using UiO-66@ Al_2_O_3_/Activated Carbon Nanocomposites. J. Inorg. Organomet. Polym. Mater..

[cit32] Wang J., Zhong K., Zeng X., Mao Y., Chen J., Nie F., Gou H., Wang J. (2025). Interfacial oxidation of boron proceeds through a stable B_6_O intermediate. Nat. Commun..

[cit33] Zhong W., Zhao H., Yao B., Li Z., Zhou S., Wang Z., Geng Y., Sun W., Fu J. (2025). Highly reconfigurable neuronlike conductive networks through nanophase structure engineering. Nat. Commun..

[cit34] Li Y., Gao P., Bai Z., Zhu W., Ding S., Zhang Y., Wang Q. (2026). Unveiling the mechanism of selective dissolution-repassivation for effective oxide film removal from alloy 690 in alkaline H_2_O_2_ solutions. Chem. Eng. J..

[cit35] Wang S., Li G., Zheng Y., Zhao D., Tong Y., Tang Y. (2026). Performance boosting of a thermoelectric generator via impinging jet array for flue gas heat harvesting from a super-adiabatic porous combustor. Energy Convers. Manage..

[cit36] Li P., Fan W., Hu R., Lei C., Wang T., Xia Z., Huang Q. (2027). New strategy for strength and ductility synergy enhancement in Ta− 12W refractory alloy: selective laser melting+ rolling+ inter− pass electrical treatment. Int. J. Extreme Manuf..

[cit37] Wang Y., Jiang C., Le Y., Cheng B., Yu J. (2019). Hierarchical honeycomb-like Pt/NiFe-LDH/rGO nanocomposite with excellent formaldehyde decomposition activity. Chem. Eng. J..

[cit38] Xu M., Zheng J., Tremblay P.-L., Jiang X., He C., Wang Y., Joya M. B., Zhang T. (2025). Enhanced photocatalytic H_2_ evolution and biological stimulation via the calcination of bulk g-C_3_N_4_ under different gases. Int. J. Hydrog. Energy..

[cit39] Chang H., Shu J., Zhu Y.-R., Yi T.-F. (2026). Synergistic modulation of electronic structure and morphology via Fe doping for high-performance bifunctional oxygen electrocatalysis. J. Energy Storage.

[cit40] Fang B., Han Y., Song Y., Shi T., Zhou S., Zeng R., Wang P. (2026). Carbonation of calcined Red Mud: Multifactor influences, reaction mechanism
and environmental impact assessment. Int. J. Greenh. Gas Control.

[cit41] Hu K., Liu A., Li R., Zheng R., Liu C., Ali M. (2026). H_2_ adsorption on coal considering the nanopore size distribution: Insight from the lattice density functional theory. J. Energy Storage.

[cit42] Chen L., Huang K., Xie Q., Lam S. M., Sin J. C., Su T., Ji H., Qin Z. (2021). The enhancement of photocatalytic CO_2_ reduction by the in situ growth of TiO_2_ on Ti_3_ C_2_ MXene. Catal. Sci. Technol..

[cit43] Madi M., Tahir M. (2022). Fabricating V_2_AlC/g-C_3_N_4_ nanocomposite with MAX as electron moderator for promoting photocatalytic CO_2_-CH_4_ reforming to CO/H_2_. Int. J. Energy Res..

[cit44] Barsoum M., Salama I., El-Raghy T., Golczewski J., Seifert H., Aldinger F., Porter W., Wang H. (2002). Thermal and electrical properties of Nb_2_AlC,(Ti, Nb)_2_AlC and Ti_2_AlC. Metall. Mater. Trans. A.

[cit45] Yang L., Du J., Deng J., Sulaiman N. H., Feng X., Liu C., Zhou X. (2024). Defective Nb_2_C MXene cocatalyst on TiO_2_ microsphere for enhanced photocatalytic CO_2_ conversion to methane. Small.

[cit46] Li W., Yue F., Shi M., Zhang L., Zhang S., Xie Y., Liu F., Zhou W., Chao H., Tang S. (2025). Thermally assisted photocatalytic industrial flue gas CO_2_ conversion: 100% selective CO production via synergistic adsorption–conversion in NH_2_–MXene–MOF hierarchical interfaces. J. Mater. Chem. A.

[cit47] Meng Y., Li W., Zamponi S., Ma Y., Zhou W., Chao H., Tang S., Berrettoni M., Zhang H. (2026). Low-temperature photothermal CO_2_-to-CO conversion from flue gas using a g-C_3_N_4_/TiO_2_/MXene heterojunction with 100% selectivity. Green Chem..

[cit48] Deng Y., Sun Y., Xu Y., Li J., Song J., Qiu J., Lei J. (2026). Preparation of nitrogen-doped TiO_2_–Ti_3_C_2_ MXene composites and their application in photocatalytic reduction of CO_2_. New J. Chem..

[cit49] Mego K., Accardo E., Ruiz-Campos P., Baldoví H. G., Atienzar P. (2026). CsPbBr_3_ nanocrystals supported on a partially oxidized Ti_2_N MXene for photothermal CO_2_ conversion. Mater. Adv..

[cit50] Du J., Deng J., Ali S., Zhou C.-A., Yue H., Schmuki P., Zhou X. (2026). Defective Nb_2_C MXene anchored Cu sites enhancing photocatalytic CO_2_ reduction to ethanol. Nanoscale.

[cit51] Albo J., García G. (2021). Enhanced visible-light photoreduction of CO_2_ to methanol over Mo_2_C/TiO_2_ surfaces in an optofluidic microreactor. React. Chem. Eng..

[cit52] Li J., Xie S., Zhu W., Di W., Gong L. (2025). Self-powered ammonia gas sensor based on Ta_2_O_5_/Nb_2_AlC nanocomposites for environmental monitoring in vegetable greenhouses. J. Environ. Chem. Eng..

[cit53] Din Babar Z. U., Fatheema J., Arif N., Anwar M., Gul S., Iqbal M., Rizwan S. (2020). Magnetic phase transition from paramagnetic in Nb_2_AlC-MAX to superconductivity-like diamagnetic in Nb_2_C-MXene: an experimental and computational analysis. RSC Adv..

[cit54] Liccardo L., Bordin M., Sheverdyaeva P. M., Belli M., Moras P., Vomiero A., Moretti E. (2023). Surface defect engineering in colored TiO_2_ hollow spheres toward efficient photocatalysis. Adv. Funct. Mater..

[cit55] Agrawal M., Chakraborty T., Yadav D., Saxena S., Shukla S. (2025). Hydrophilic and superoleophobic GO-TiO_2_ PVDF/PEI membrane with antifouling properties for efficient oil-water separation. Next Mater..

[cit56] Murthy Sridevi V., Natarajan A., Rajaram A., Suresh P., Rajagopal K. (2024). Investigation of Nb_2_AlC Max Phase Integrated With 2D GCN (Nb-MX/GCN) as a Bifunctional Electrocatalyst for Water Splitting Reaction. ChemistrySelect.

[cit57] Tahir B., Tahir M., Nawawi M. G. M. (2022). Highly stable honeycomb structured 2D/2D vanadium aluminum carbide MAX coupled g-C_3_N_4_ composite for stimulating photocatalytic CO_2_ reduction to CO and CH_4_ in a monolith photoreactor. J. Alloys Compd..

[cit58] Landi Jr S., Segundo I. R., Afonso C., Lima Jr O., Costa M. F., Freitas E., Carneiro J. (2022). Evaluation of band gap energy of TiO_2_ precipitated from titanium sulphate. Phys. Rev. B.

[cit59] Murphy A. (2007). Band-gap determination from diffuse reflectance measurements of semiconductor films, and application to photoelectrochemical water-splitting. Sol. Energy Mater. Sol. Cells.

[cit60] Lettieri S., Pavone M., Fioravanti A., Santamaria Amato L., Maddalena P. (2021). Charge carrier processes and optical properties in TiO_2_ and TiO_2_-based heterojunction photocatalysts: A review. Materials.

[cit61] Shi J. , Wang X., Feng Z., Chen T., Chen J. and Li C., Photoluminescence spectroscopic studies on TiO_2_ photocatalyst, Environmentally Benign Photocatalysts: Applications of Titanium Oxide-based Materials, Springer, 2010, pp. 185–203

[cit62] Li J., Kuang D., Feng Y., Zhang F., Liu M. (2012). Glucose biosensor based on glucose oxidase immobilized on a nanofilm composed of mesoporous hydroxyapatite, titanium dioxide, and modified with multi-walled carbon nanotubes. Microchim. Acta.

[cit63] Sharma S., Sharma P., Mittal A., Sharma A., Motaung D. E., Chauhan N. S., Kumar N. (2023). Development of TiO_2_/Bi_2_O_3_/PANI as a novel glucose biosensor and antimicrobial agent. Inorg. Chem. Commun..

[cit64] Suliman M., Tahir M. (2026). Engineering MAB mediated In^3+^/MoAlB/TiO_2_ nanocomposite with synergistic effect of In^3+^/MAB for solar driven seawater splitting to produce hydrogen. Int. J. Hydrogen Energy.

[cit65] Mittal A., Sharma S., Kumari V., Yadav S., Chauhan N. S., Kumar N. (2019). Highly efficient, visible active TiO_2_/CdS/ZnS photocatalyst, study of activity in an ultra low energy consumption LED based photo reactor. J. Mater. Sci.: Mater. Electron..

[cit66] Adnan M., Zhao Z., Ashraf G. A., Bashir S., Teng B., Yuennan J., Alotaibi S. S., Mushtaq M. (2025). Novel MAX-phase Nb_2_AlC@ Biochar: A dual-functional peroxymonosulfates activator for antibiotics and dyes degradation via Nb^4+^/Nb^5+^ redox switches; DFT insight and mechanistic elicitation. J. Water Process Eng..

[cit67] Tahir M., Tahir B., Kumar N., Al Marzooqi M., Siraj M., Fatehmulla A. (2024). In-situ synthesis of V_2_AlC@ V_2_O_5_/TiO_2_ immobilized over honeycomb support with vanadium oxide electron transfer mediator for stimulating selective CO_2_ photoreduction through bi-reforming in a monolith reactor. Fuel.

[cit68] Tang Q., Sun Z., Deng S., Wang H., Wu Z. (2020). Decorating g-C_3_N_4_ with alkalinized Ti_3_C_2_ MXene for promoted photocatalytic CO_2_ reduction performance. J. Colloid Interface Sci..

[cit69] Mei W., Zhao F., Xu K., Xia W., Zhang J., Yu J., García H., Xu F. (2026). Schottky− Defect–Photothermal Coupling in Nb_2_C/Nb_2_O_5–x_ Heterostructures for Enhanced CO_2_ Photoreduction. ACS Catal..

[cit70] Liu T., Tan G., Feng S., Zhang B., Liu Y., Wang Z., Bi Y., Yang Q., Xia A., Liu W. (2023). Dual localized surface plasmon resonance effect enhances Nb_2_AlC/Nb_2_C MXene thermally coupled photocatalytic reduction of CO_2_ hydrogenation activity. J. Colloid Interface Sci..

[cit71] Karamian E., Sharifnia S. (2016). On the general mechanism of photocatalytic reduction of CO_2_. J. CO_2_ Util..

[cit72] Li Q., Wang H., Zhang M., Li G., Chen J., Jia H. (2023). Suppressive strong metal-support interactions on ruthenium/TiO_2_ promote light-driven photothermal CO_2_ reduction with methane. Angew. Chem., Int. Ed..

[cit73] Zhang L., Li C., Liu Y., Xu C., Zhang Y. (2024). Unraveling active sites regulation and temperature-dependent thermodynamic mechanism in photothermocatalytic CO_2_ conversion with H_2_O. Npj Comput. Mater..

[cit74] Novoa-Cid M., Baldovi H. G. (2020). Study of the photothermal catalytic mechanism of CO_2_ reduction to CH_4_ by ruthenium nanoparticles supported on titanate nanotubes. Nanomaterials.

[cit75] Li G., Zakharov D. N., Hu T., Yu Y., Waluyo I., Hunt A., Head A. R., Boscoboinik J. A. (2024). Tracking the dynamics of catalytic Pt/CeO_2_ active sites during water-gas-shift reaction. Commun. Mater..

[cit76] Wang Y., Zhao J., Wang T., Li Y., Li X., Yin J., Wang C. (2016). CO_2_ photoreduction with H_2_O vapor on highly dispersed CeO_2_/TiO_2_ catalysts: Surface species and their reactivity. J. Catal..

[cit77] Cai S., Zhang M., Li J., Chen J., Jia H. (2021). Anchoring single-atom Ru on CdS with enhanced CO_2_ capture and charge accumulation for high selectivity of photothermocatalytic CO_2_ reduction to solar fuels. Sol. RRL.

[cit78] Shangguan W., Liu Q., Wang Y., Sun N., Liu Y., Zhao R., Li Y., Wang C., Zhao J. (2022). Molecular-level insight into photocatalytic CO_2_ reduction with H_2_O over Au nanoparticles by interband transitions. Nat. Commun..

[cit79] Poudyal S., Laursen S. (2018). Insights into elevated-temperature photocatalytic reduction of CO_2_ by H_2_O. J. Phys. Chem. C.

[cit80] Tahir M. (2023). Photocatalytic CO_2_ Reduction to Produce Cleaner Fuels Over Vanadium Aluminium Carbide (V_2_AlC) Supported TiO_2_ Composite. Chem. Eng. Trans..

[cit81] Tahir M. (2020). Enhanced photocatalytic CO_2_ reduction to fuels through bireforming of methane over structured 3D MAX Ti_3_AlC_2_/TiO_2_ heterojunction in a monolith photoreactor. J. CO_2_ Util..

[cit82] Wang L., Qiu J., Wu N., Yu X., An X. (2023). TiO_2_/CsPbBr_3_ S-scheme heterojunctions with highly improved CO_2_ photoreduction activity through facet-induced Fermi level modulation. J. Colloid Interface Sci..

[cit83] Binxia Yuan H. Q., Luo Z., Zhu R. (2023). Weiling Luan A green synthetic approach for C/Ag@ urchin-like TiO_2_ nanocomposites showing a highly molar ratio CH_4_/CO for CO_2_ photoreduction. Mater. Lett..

[cit84] Ren G., Wei Z., Li Z., Zhang X., Meng X. (2023). Fabrication of S-scheme hollow TiO_2_@Bi_2_MoO_6_ composite for efficiently photocatalytic CO_2_ reduction. Mater. Today Chem..

[cit85] Merino-Garcia I., García G., Hernández I., Albo J. (2023). An optofluidic planar microreactor with photoactive Cu_2_O/Mo_2_C/TiO_2_ heterostructures for enhanced visible light-driven CO_2_ conversion to methanol. J. CO_2_ Util..

[cit86] Ijaz N., Jalal F. E., Jaffar S. T. A., Alshameri B., Ijaz Z., Zhou N. (2026). Mechanistic insights into mineral-specific interactions in biopolymer-stabilized soils. J. Rock Mech. Geotech. Eng..

