# Supplementary material for: Wavelength-controlled photothermal reduction of carbon dioxide to produce fuels over Nb2AlC/TiO2 heterostructures in a continuous-flow reactor
Source: RSC Adv. 2026 Nov 11. Online ahead of print. doi: 10.1039/d6ra09084b (PMC13753979; doi:10.1039/d6ra09084b)
Supplement: Supplementary file 1 [file RA-OLF-D6RA09084B-s001.pdf]

Supplementary Information (SI) for RSC Advances.
This journal is © The Royal Society of Chemistry

Wavelength-Controlled Photothermal Reduction of Carbon Dioxide to Produce Fuels Over Nb_2AlC/TiO_2 Heterostructures in a Continuous-Flow Reactor

Anuradha Sharma[a], Muhammad Tahir[a*], Naveen Kumar[b]

[a] Chemical and Petroleum Engineering Department, United Arab Emirates University, Al Ain 15551, United Arab Emirates.

[b] Department of Chemistry, Maharshi Dayanand University, Rohtak, 124001, India.

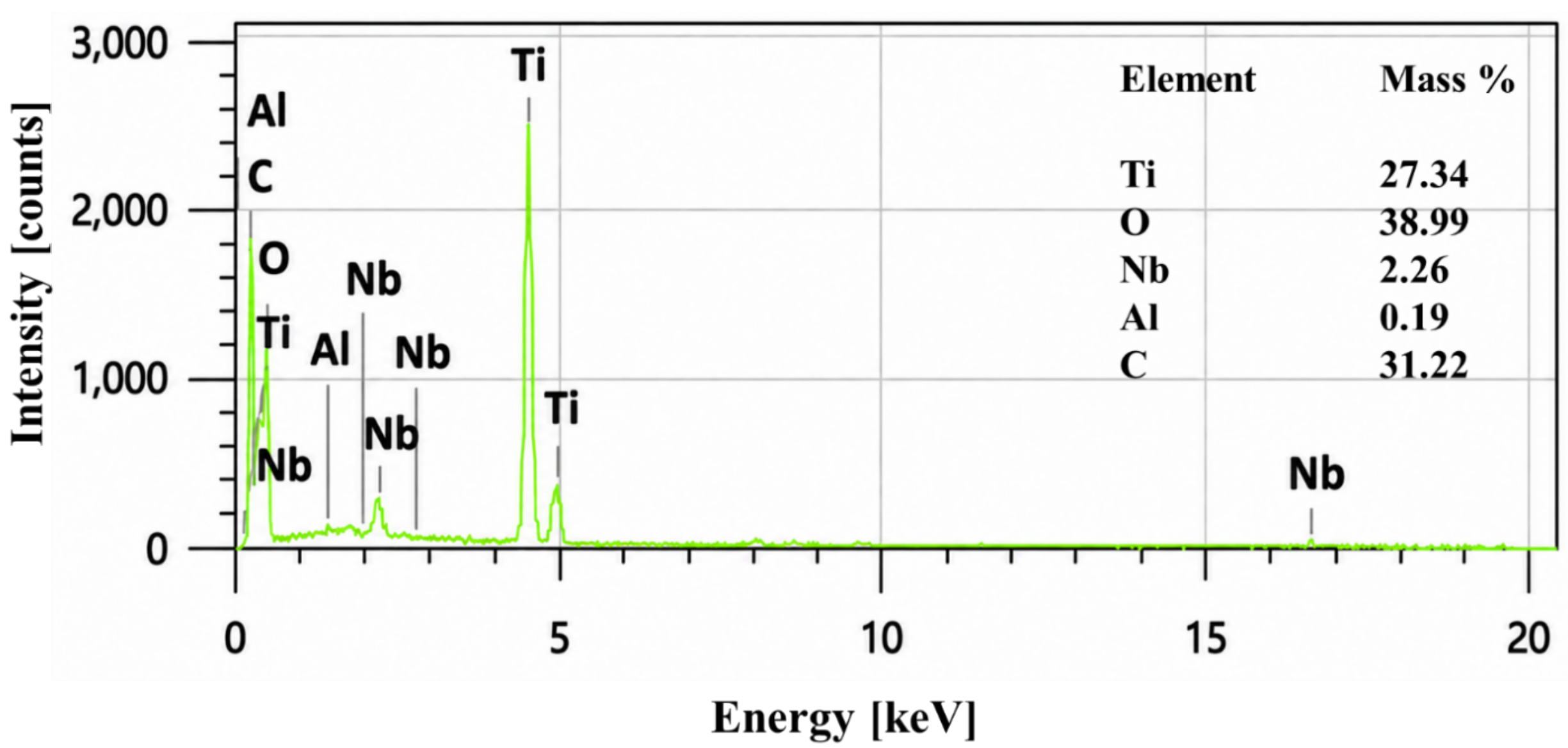

Fig. S1 EDS spectrum of the 10-Nb_2AlC/TiO_2 composite
